# Cortical layer-specific abnormalities in auditory responses in a mouse model of Fragile X Syndrome

**DOI:** 10.1016/j.nbd.2025.106963

**Published:** 2025-05-18

**Authors:** Katrina E. Deane, Devin K. Binder, Khaleel A. Razak

**Affiliations:** a Psychology Department, University of California, Riverside, 900 University Ave, Riverside, CA 92521, USA; b Graduate Neuroscience Program, University of California, Riverside, 900 University Ave, Riverside, CA 92521, USA; c Biomedical Sciences, School of Medicine, University of California, Riverside, 900 University Ave, Riverside, CA 92521, USA

**Keywords:** Autism spectrum disorders, Fragile X Syndrome, Temporal processing, Sensory hypersensitivity, Laminar processing, Primary auditory cortex

## Abstract

Fragile X Syndrome (FXS) is a leading genetic cause of autism spectrum disorders (ASD)- associated behaviors, including sensory processing deficits. Sensory sensitivity and temporal processing deficits in the auditory domain will affect development of language and cognitive functions. The mouse model for FXS, *Fmr1* KO, has shown remarkably similar auditory processing phenotypes to patients with FXS. In vitro cortical slice recordings show layer-specific differences in *Fmr1* KO mouse local circuits, but it is unclear how these differences translate to changes in sensory processing. In this study, we used a depth multielectrode to record in vivo spikes and local field potentials across layers of the auditory cortex in *Fmr1* KO and wildtype mice (WT), converting the latter to current source density (CSD) profiles for improved spatial resolution analysis. We observed reduced CSD sink amplitudes and inter-trial phase coherence, and an increase in trial-to-trial variability for temporally modulated stimuli in the KO mice. Results indicated a differential cortical layer pattern of activity in KO mice, with higher baseline gamma power in superficial and deep layers and higher resting delta and theta power in granular layers. Significantly elevated inter-trial variability was observed for CSD and spikes in KO mice. Auditory steady state responses to clicks or gaps at 40 Hz showed considerable trial-to-trial variability in a layer-specific manner in KO mice. Neural generators in the *Fmr1* KO mouse auditory cortex failed to detect short gaps in noise, indicating severe temporal processing deficits. Altogether, this study indicates layer-specific cortical mechanisms of sensory hypersensitivity and temporal processing deficits in FXS.

## 1. Introduction

Autism Spectrum Disorders (ASD) encompass a wide range of debilitating symptoms, including a consistent association with sensory deficits and abnormal language development. Sensory sensitivity and temporal processing deficits in ASD may lead to language dysfunction, but the underlying mechanisms are unclear (Cabrera and Gervain, 2020; Foss-Feig et al., 2017; Rapin and Dunn, 2003; Shannon et al., 1995; Tager-Flusberg and Caronna, 2007; Zeng et al., 2005). Layer-specific differences in local cortical circuit function and connectivity differences are reported in ASD rodent models (Contractor et al., 2015; Falcone et al., 2021; Goswami et al., 2019; Krishnan et al., 2015; Kroon et al., 2019; Liu et al., 2020; Rotaru et al., 2018). But how cortical laminar dynamics and layer differences may lead to in vivo sensory processing differences remain unclear in humans with ASD or any ASD animal model.

The main goal of the current study is to quantify layer-specific differences in auditory processing in a mouse model of Fragile X Syndrome (FXS), the *Fmr1* knock out (KO), compared to wildtype (WT) counterparts. FXS is the leading known genetic cause of intellectual deficits and ASD-associated behaviors including social and anxiety differences, repetitive behaviors, sensory, language and learning delays (Hagerman et al., 2017). FXS affects up to 1 in 4000 males and 1 in 7000 females, and results from the silencing of the Fragile X Messenger Ribonucleoprotein (*Fmr1*) gene on the X chromosome. This leads to a partial or complete loss of the Fragile X Messenger Ribonucleoprotein (FMRP). FMRP is as an RNA binding protein in the brain and other tissues, and consequences of the loss of FMRP include abnormal protein synthesis, synaptic development and plasticity (Richter and Zhao, 2021).

Clinical, behavioral and electrophysiological studies demonstrate sensory hypersensitivity in humans with FXS across multiple domains (Miller et al., 1999; Rais et al., 2018; Sinclair et al., 2017). This is particularly consistent and debilitating in the auditory system (Razak et al., 2021). Electroencephalographic (EEG) recordings in humans with FXS suggest that cortical hyperexcitability likely underlies auditory hypersensitivity behavior. Correlates of hyperexcitability include elevated evoked response amplitudes, reduced habituation of responses, increased gamma band power in resting EEG, increased single trial power and abnormal phase locking fidelity to dynamic auditory stimuli (Razak et al., 2021). Of important translational relevance, *Fmr1* KO mouse and rat models also show auditory hyperexcitability and hypersensitivity (Auerbach et al., 2021; Kozono et al., 2020; Lovelace et al., 2018; Rotschafer and Razak, 2013). Single neuron recordings from the auditory cortex show increased response magnitude, broader frequency tuning curves and greater variability of responses across trials (Rotschafer and Razak, 2013, 2014). EEG recordings from awake mice show remarkably similar phenotypes as seen in humans, including higher resting gamma power, higher single trial power, larger event related potential (ERP) amplitudes, reduced habituation of responses and reduced phase locking to dynamic stimuli (Kozono et al., 2020; Lovelace et al., 2018).

In vitro, Goswami et al. (2019) found higher spike activity in layer V of the auditory cortex of *Fmr1* KO mice and higher inter-layer spike correlation between layers II/III and V, when layers II/III were stimulated. This suggests that at least some mechanisms underlying hyperexcitability includes local cortical processing deficits. This is supported by findings that deletion of FMRP from only the forebrain recapitulates most, but not all, EEG deficits seen in global *Fmr1* KO mice (Lovelace et al., 2020b). One method to link local circuit function that can be determined in rodents to macroscopic EEG type recordings in humans is by recording local field potentials (LFP) and quantifying current source density (CSD) across cortical layers (Mitzdorf, 1985; Pitts et al., 1952), which facilitate an analysis of local subthreshold activity in specific cortical layers (Deane et al., 2024; Happel et al., 2014; Mitzdorf, 1985). However, in vivo layer-specific cortical processing in *Fmr1* KO mice, or in other ASD models, is unclear. Layer-specific processing deficits will lead to testable hypotheses about the links between local circuit function and EEG phenotypes. Therefore, we investigated laminar responses in the primary auditory cortex (A1) of urethane-xylazine anesthetized C57bl6/J wildtype (WT) and *Fmr1* KO mice in response to similar sound stimulation paradigms as used in previous mouse and human FXS EEG studies. LFP data were recorded across layers of A1 and transformed into CSD profiles. In addition, multiunit spiking activity was recorded from the same electrodes to compare LFP and spiking responses across layers. Based on these recordings, we compared CSD magnitudes at rest and in response to noise bursts, temporal processing using 40 Hz auditory steady state responses, and gap-in-noise stimuli. We report multiple novel layer-specific auditory processing differences between WT and *Fmr1* KO mice.

## 2. Materials and methods

### 2.1. Mice

All procedures were approved by the Institutional Animal Care and Use Committee at the University of California, Riverside and were done in accordance with the NIH Animal Care and Use Guidelines. The mice used in this study were non-littermate male wild-type (WT, *n* = 10, postnatal day (p)60–90) and *Fmr1* KO mice (*n* = 9, p60–90) on a C57bl6/J background maintained in-house. The colony was established from breeding pairs of *Fmr1* KO (B6.129P2-Fmr1tm1Cgr/J, stock #003025) and C57BL/6 J WT (stock #000664) from Jackson Laboratory (Bar Harbor, ME). Mice were group-housed with two to five mice per cage in an AAALAC-accredited facility under a 12-h light/dark cycle and were provided irradiated rodent diet (PicoLab, 5053) and water ad libitum. All genotypes were confirmed by Transnetyx (Cordova, TN). The C57bl6/J strain of mice were chosen as the majority of EEG studies on *Fmr1* KO mice that showed similarities to human phenotypes have been performed in this background. We recorded auditory responses before the onset of functional hearing loss in this strain of mice (Mikaelian et al., 1974).

### 2.2. Surgery and probe implantation

*Fmr1* KO or WT mice were anesthetized with urethane/xylazine anesthesia (1/10 mg/kg) intraperitoneally. The left auditory cortex was exposed by craniotomy and the primary auditory cortex (A1) was located by vascular landmarks, and coordinates from previous single unit recording studies (Rotschafer and Razak, 2013). A small hole was drilled over the right hemisphere, above the visual cortex, for implanting a stainless-steel reference wire (Ø 200 μm) and on the left hemisphere for a ground wire. Mice were moved to an anechoic chamber (Gretch-Ken, OR) and head-fixed via a bar fastened to the medial skull with dental cement. A 32-channel vertical array probe (A1x32-6mm-50-177, Neuronexus, Ann Arbor, Michigan USA) was attached to an electrode micro-manipulator with x, y, z and angle adjustment (Model 960, David Kopf Instruments, Tujunga, CA). The angle was adjusted to the plane of the A1 relative to the head-fixation and the probe was inserted perpendicularly into the A1 for acute recordings. This was facilitated by the lissencephalic nature of the mouse neocortex. The perpendicular angle was confirmed by consistency of best frequency across depth in a few of the recordings as detailed below. During insertion, 1–10 channels were left above the cortical surface to ensure proper depth. Layer IV thalamic input is marked by the channel with the lowest delay of sound-induced response (detailed further below). Mice were kept on a heating pad during the procedure to a body temperature of 37 ^◦^C and the state of anesthesia was checked every 10–20 min by toe-pinch reflex. Additional doses of the anesthetic mixture at ~quarter strength were given as necessary.

### 2.3. Electrophysiological recordings

Auditory stimuli were generated using RPvdsEX software (Tucker Davis Technologies (TDT)) through an RZ6 (TDT) multi-I/O processor and presented using an open-field MF1 speaker (TDT) located 25 cm distant from and at the same height as the right ear. A ¼” free-field microphone (type 4939) and Nexus conditioning amplifier (type 2690; both Hottinger Brüel & Kjær, London, United Kingdom) were used to calibrate acoustic stimuli. Neural activity was recorded with XDAQ One (NeuroNexus) and fed into Allego recording software (NeuroNexus). Signals were then low-pass filtered (200 Hz) for local field potential (LFP) activity and bandpass filtered (300–5000 Hz) for spikes via Curate (NeuroNexus) and converted to .mat files for analysis in Matlab (2023, Mathworks).

A series of auditory stimuli were presented to each subject:

Noise-bursts: 100 ms stimulus duration, 1000 ms inter-trial-interval (ITI), 50 pseudorandomized repetitions across each sound pressure level (SPL): 20, 30, 40, 50, 60, 70, 80, and 90 dB.

Click trains: train duration of 2000 ms, ITI of 2000 ms, 70 dB SPL, 50 pseudorandomized repetitions across each click rate: 2, 5, 10, 20, 40, and 80 Hz. The 40 Hz click train is typically used to measure 40 Hz auditory steady state response (ASSR).

Gap-in-noise ASSR stimuli: This is a modified 40 Hz ASSR stimulus in which short gaps with different gap widths are introduced to study temporal acuity. Stimulus duration of 3800 ms, ITI of 400 ms, 70 dB SPL, alternating 250 ms blocks of noise without gaps (7 blocks) and gap-in-noise (6 blocks) where ‘gaps’ are at 75 % modulation depth repeated at 40 Hz, 50 pseudorandomized repetitions across each gap width: 2, 3, 6, 8, and 10 ms (for more details of gap-ASSR paradigm, Rumschlag and Razak, 2021).

Tone responses: To confirm that the depth probe was inserted perpendicular to A1, we tested whether BF was consistent across the cortical column, which should generally be the case if the electrode was inserted perpendicular to the plane of A1. Pure tones of 200 ms duration, with 10 ms rise and fall times, and at octave intervals (1, 2, 4, 8, 16, 24, and 32 kHz) were pseudo-randomly presented at 70 dB SPL. Peak amplitude from the average sink activity per layer as detailed below was detected from the first 60 ms of pure tone presentation. Supplementary Fig. 1 shows the peak amplitude tuning curves and best frequency (BF) for each layer of the cortex for 2 example subjects from each group. In each of these mice, there was consistency in the BF across the layers, suggesting recordings from a cortical column and confirming perpendicular orientation of the probe.

Spontaneous: Two minutes of spontaneous neural activity was also recorded per subject.

### 2.4. Current source density analysis

Based on the recorded laminar LFPs, the second spatial derivative was calculated in Matlab (R2016a-R2022a), yielding the CSD distribution (eq. 1):

(1) $$CSD\approx \frac{\delta^{2}\Phi (z)}{\delta z^{2}}=\frac{\Phi (z+n\Delta z)-2\Phi (z)+\Phi (z-n\Delta z)}{(n\Delta z{)}^{2}}$$

where *Φ* is the field potential, *z* is the spatial coordinate perpendicular to the cortical laminae, Δ*z* is the sampling interval, and *n* is the differential grid (Mitzdorf, 1985). LFP profiles were smoothed with a weighted average (Hamming window) of 9 channels which corresponds to a spatial kernel filter of 450 μm (Happel et al., 2010). CSD distributions reflect the local spatiotemporal current flow of positive ions from extracellular to intracellular space evoked by synaptic activity in laminar neuronal structures. Current sinks thereby correspond to the activity of excitatory synaptic populations, while current sources reflect balancing return currents. Early synaptic thalamocortical inputs persist after intracortical silencing with the GABAA-agonist muscimol related to thalamocortical projections on cortical layers III/IV and Vb/VIa (Brunk et al., 2019; Deane et al., 2020; Happel et al., 2010, 2014; Happel and Ohl, 2017) in accordance with reports by others (Schaefer et al., 2015). Early current sinks in the auditory cortex are therefore indicative of thalamic input in granular layers III/IV and infragranular layers Vb/VIa (Happel et al., 2010; Szymanski et al., 2009). Cortical layer designations were made in consideration of previous auditory cortex laminar work in the house mouse (Deane et al., 2024; Muramatsu et al., 2019; Yamamura et al., 2017) and Mongolian gerbil (Deane et al., 2020; Happel et al., 2010, 2014). Briefly, the mouse A1 is ~1 mm deep, fitting ~20 channels (50 μm apart). Channels above and below the cortex were identifiable due to larger and consistent slow oscillations in white matter, below, and high noise in the air, above. Channels were assigned to layers based on consistent rodent CSD A1 sink-source profiles (see citations above), with a superficial layer (I), granular thalamic input and immediate recurrent excitation (II-IV), and above (Va), within (Vb), and below (VI) the infragranular thalamic input layer. All laminar analysis either averaged across assigned layer channels per subject or took the center channel (center ceiling in the case of an even number of channels). This controlled for slight variability in layer depth across subjects (Supplementary Figs. 2 and 3).

CSD profiles were further transformed by averaging the rectified waveforms of each channel by eq. 2:

(2) $$AVREC=\frac{\sum_{i=1}^{n}\left|CSD_{i}\right|(t)}{n}$$

where *n* is the individual channel and *t* is time in ms. This measure gives us the overall temporal local current flow of the columnar activity (Givre et al., 1994; Schroeder et al., 1998). Layer traces were calculated by averaging channel activity in each layer after replacing source activity (positive) with NaN, leaving only sink activity (negative), and then multiplying by –1. After AVREC and layer traces were calculated, they were normalized per measurement. For layer analysis, each subjects’ individual layer designations were taken, either fully (as in Layer Traces) or just their center channel (as in spectral analysis, below).

### 2.5. Spectral analysis

#### 2.5.1. Spontaneous fast fourier transform

Fast Fourier transform (FFT) was calculated for each subjec’s 2-min spontaneous neural activity recording. Spectral power was computed with eq. 3:

(3) $$\text{Power}=\left|a+b_{i}\right|2$$

where *a* + *b_i_* represents the complex number output of the FFT analysis. Each subjec’s power spectrum was further normalized by the mean of the WT group. Frequency bands analyzed were delta 1–4 Hz, theta 4–7 Hz, alpha 8–12 Hz, beta 13–30 Hz, low gamma 31–60 Hz, and high gamma 61–100 Hz.

#### 2.5.2. Continuous wavelet transform analysis

Spectral analysis was performed in Matlab using the wavelet analysis toolbox function *CWT* (continuous wavelet transform) for the following variables: animal, condition, stimulus, and recorded signal. Important parameters fed into the CWT were as follows: layer channels from CSD profiles, frequency limits: 5 to 100 Hz (below the Nyquist), and wavelet used: analytic Morse (Lilly and Olhede, 2012; Olhede and Walden, 2002). For layer-wise wavelet analysis, the center channel of each layer was fed into the CWT. Single trial scalograms were calculated for each measurement and inter-trial phase coherence (ITPC) for each subject was computed with eq. 4:

(4) $$\mathrm{ITPC}=\left|\frac{\sum \left(a+b_{i}\right)/\left|a+b_{i}\right|}{n}\right|$$

where *a* + *b_i_* represents the complex number output of the trial-averaged CWT analysis. The ITPC measures the phase consistency of the recorded signal across multiple trials. The ITPC is based on the distribution of phase angles in the response at 40 Hz across all trials and reflects the precise timing of 40 Hz activity in the underlying LFP/CSD neural generators.

#### 2.5.3. Spike detection

Multi-unit spike detection was algorithmically computed with Videre (NeuroNexus) after a band-passed filter (300–3000 Hz) was applied to the raw, 30,000 sampling frequency data. Videre settings were as follows: detection threshold: −4 standard deviations from the baseline, feature dimension: 1, position influence: 0.5, waveform influence: 0.5. Channel locations and times of spikes were stored per trial, per subject, so that raster plots and peristimulus time histograms (PSTH) could be generated across subjects and groups over layers. For PSTH plots, spike count over time was averaged over trials, normalized by number of channels assigned to cortical layer, then averaged over subjects. Spike rate was calculated in each channel as the number of spikes per trial over time. Fano factor was calculated in each channel with eq. 5.

(5) $$F(t)=\frac{\sigma_{t}^{2}}{\mu_{t}}$$

where σ is the standard deviation and μ is the mean number of spikes over time (*t*). Spike rate and Fano factor were then averaged over channels per cortical layer.

### 2.6. Statistics

Peak amplitudes, latency, and root mean square (RMS) were detected from the following time windows per stimuli: noise bursts, 0–50, 50–100, and 100–200 ms; click trains, from each click onset to the next (20 ms each); gap ASSR, from the onset to offset (250 ms) of each gap-in-noise block. Bonferroni-corrected Student’s *t-*tests and Cohen’s d effect sizes were run on this extracted data at a single-trial level. Coefficient of variance was calculated per subject and compared across groups with a Studen’s *t-*test and Cohen’s d effect size. FFT normalized power was averaged per subject for each frequency band, delta-gamma, and a Bonferroni corrected Studen’s *t*-test and Cohen’s d effect size was calculated across groups. In all cases for *t* and d tests, * = *p *< 0.05, ** = *p* < 0.01, *** = *p *< 0.001, (small) S = d < 0.5, (medium) M = 0.51 < d < 0.8, (large) L = 0.81 < d < 1.2, (very large) VL = 1.21 < d < 2, (huge) H = d > 2.

Spike rate and Fano factor were calculated over the 2 s epochs for the resting condition. For the noise burst stimulus, they were calculated at –100-0, 0–100, and 200–300 ms around stimulus onset (0 ms). For both ASSR stimuli, spike rate and Fano factor were calculated at –100-0, 0–100 around stimulus onset. They were also calculated within a time window where ASSR was sustained, 1150–1600 ms in the 40 Hz ASSR stimulus and 2150–2600 ms in the gap ASSR stimulus. These data were then compared across groups with a Studen’s *t-*test and Cohen’s d effect size.

To find group differences in ITPC, we used the non-parametric permutation clustermass analysis (Maris and Oostenveld, 2007; Rumschlag and Razak, 2021). First, a Studen’s *t-*test was run on each time-frequency point across groups, yielding point-wise *t*-values. *t*-values corresponding to two-tailed *p *< 0.025 were considered significant. Clusters of significance were traced via the Matlab function *bwboundaries*. Next, the group assignments were shuffled randomly, and the *t-*tests and cluster-measurements were run again on the permuted groups. This was performed 1000 times to generate a distribution of cluster sizes that we would expect to find by chance. In the observed group comparison, clusters that were larger than 95% of the permutation clusters were considered to be significant. Any observed cluster smaller than 3 × 3 was discarded. This method allows for the discovery of significant differences between groups while accounting for multiple comparisons, and without making assumptions about normality or homogeneity of variance between groups.

## 3. Results

### 3.1. Layer specific differences in spectral power distribution in Fmr1 KO mice

**Fig. 1.**
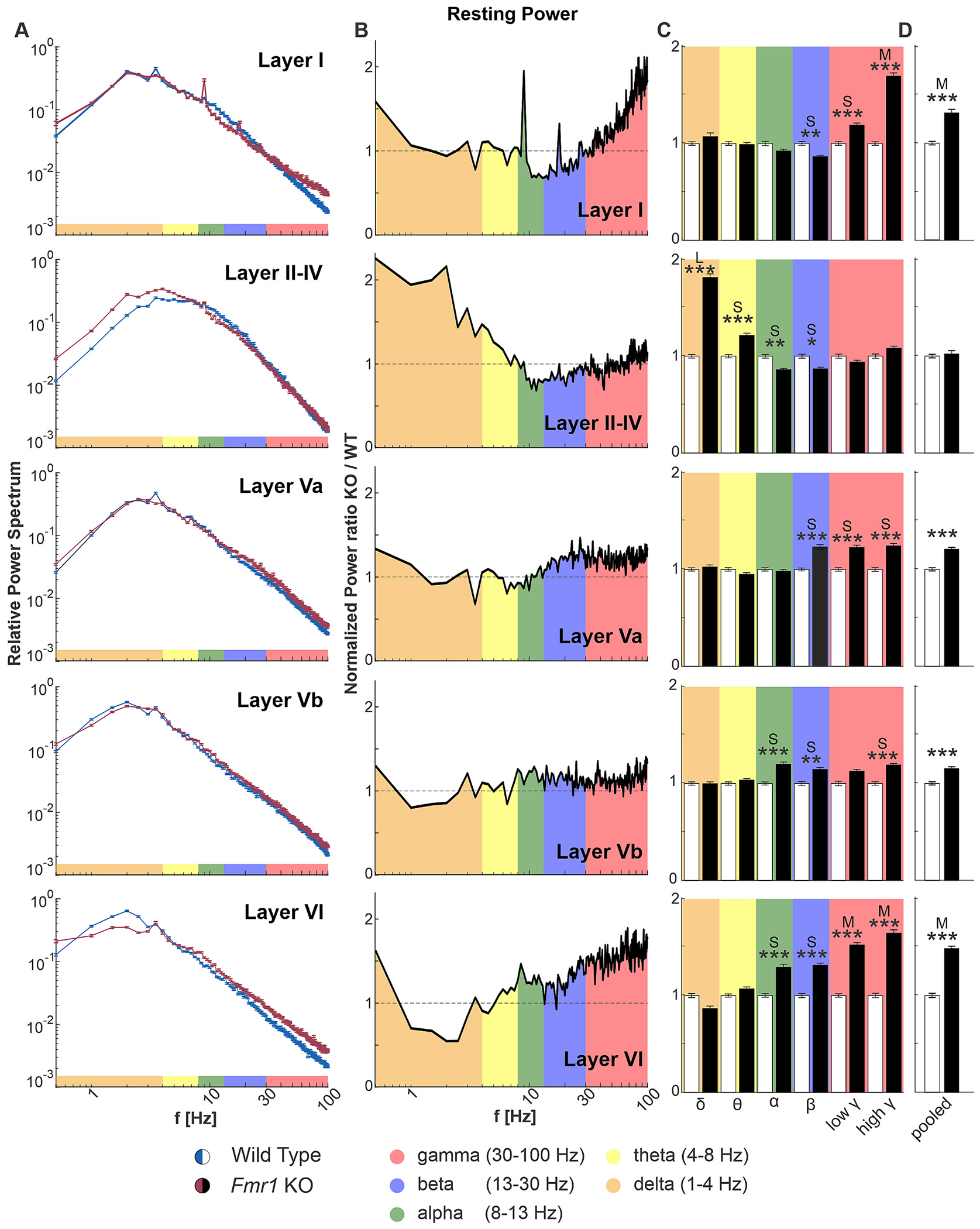
Higher resting gamma in superficial and deep layers, not in II-IV. Spontaneous data taken from 2 continuous minutes of recording with no stimuli presentation. Column A: Relative FFT power spectrum for *Fmr1* KO (red) and Wildtype (blue) mice for each layer. B: FFT power spectrum normalized by dividing each KO subject by the WT group mean within each layer. The dotted line in each graph at ‘1’ represents the WT group mean. C: FFT power bar graphs of the ratio of normalized KO subjects / WT mean (black) and WT subjects / WT mean (white) within each layer, separated by spectral band, delta-high gamma. D: FFT power bar graphs of the ratio of subjects / WT mean, pooled across the spectrum, 0–100 Hz. Colors denote each spectral band, orange = delta, yellow = theta, green = alpha, blue = beta, and red = gamma. All *t*-tests were Bonferroni-corrected; *p* < 0.05 *, *p* < 0.01 **, *p* < 0.001 ***. Cohen’s d calculated for each t-test; S (small) d > 0.2, M (medium) d > 0.5, L (large) d > 0.8, VL (very large) d > 1.2. (For interpretation of the references to colour in this figure legend, the reader is referred to the web version of this article.)

A consistent EEG phenotype seen in *Fmr1* KO mice, *Fmr1* KO rats, and in humans with FXS is the higher resting EEG gamma power (Razak et al., 2021). A few studies have also reported larger low-frequency (delta/theta) power, and reduced alpha band power, resulting in a ‘U’ shaped increase in FXS/ASD (Lovelace et al., 2018; Wang et al., 2013). To determine if there are layer-specific differences in in vivo resting power, two minutes of spontaneous cortical LFP activity was recorded from each subject using the depth probe, and FFT was used to calculate power across this time window on the center channel of each layer. The resulting power spectra (Fig. 1A) were normalized to the WT group mean across subjects (Fig. 1B). Spectral frequency bands were then separated, delta through high gamma, and WT and KO groups were compared at each spectral band (Fig. 1C, Table 1) and across pooled bands (Fig. 1D, Table 1 for full statistical results). Compared to WTs, *Fmr1* KO mice have higher resting power in non-granular layers, significant in layer I with lower beta and elevated low gamma and high gamma power, and layer VI with increased alpha, beta, low gamma, and high gamma power. With only small Cohen’s d effect sizes, layer Va has significantly higher beta, low gamma, and high gamma and layer Vb has significantly higher alpha, beta, and high gamma. In contrast, granular input and recurrent excitation layer II-IV has equivalent resting gamma across groups, significantly lower alpha and beta, but higher delta and theta power in *Fmr1* KO mice, with a large effect size for the delta band. This translates to significantly higher pooled differences in layers I and VI with medium effect sizes, as well as in layers Va and Vb but with small effect sizes. Taken together, these data reveal layer-specific differences in specific spectral bands that may explain the ‘U’ shaped change in EEG resting power in humans with FXS/ASD, compared to typically developing control subjects (Wang et al., 2013).

**Fig. 2.**
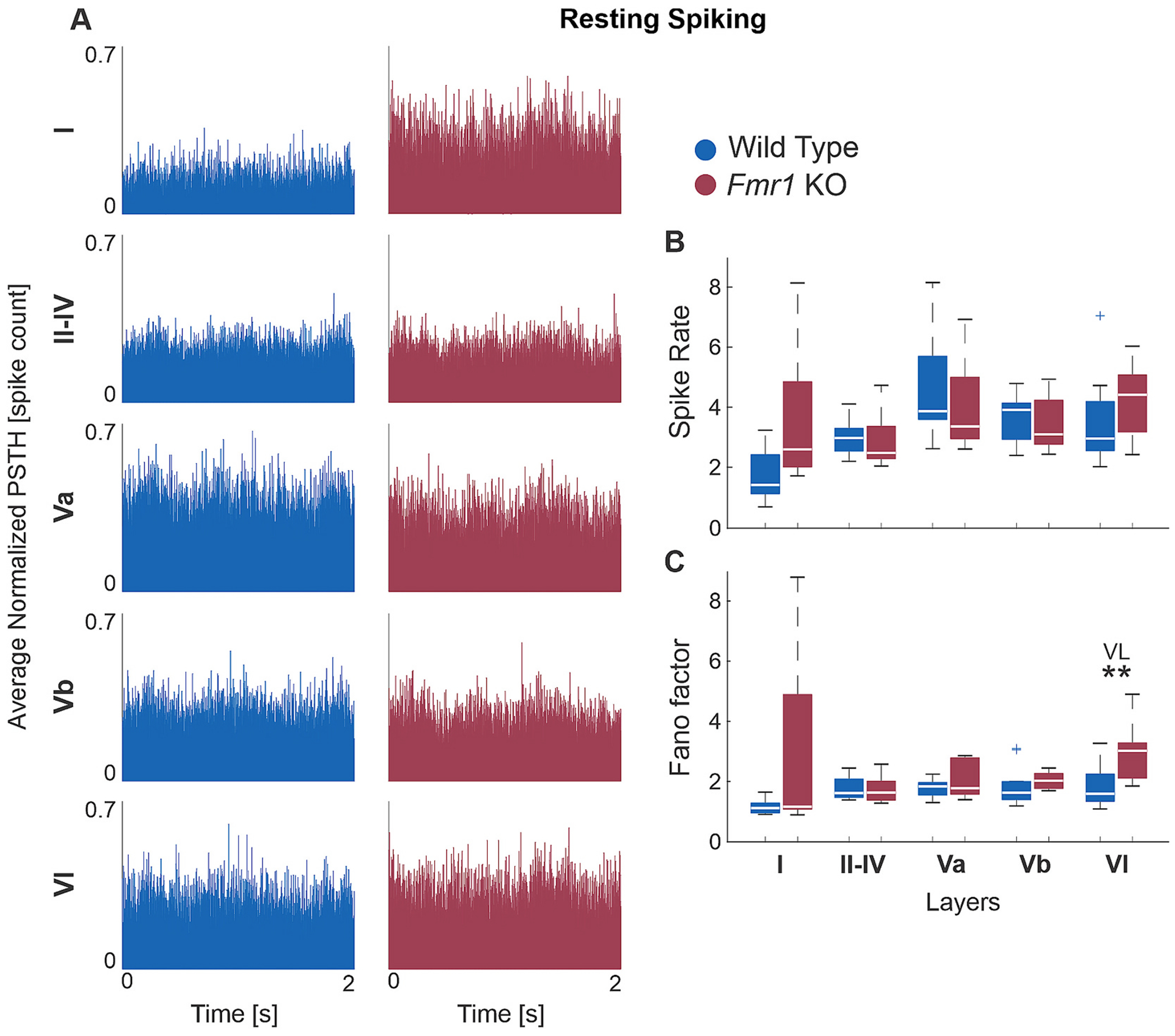
Higher spike rate and inter-spike-interval (ISI) in lower cortical layers during spontaneous spiking. A: Group-averaged PSTH, normalized by number of channels per subject layer, for WT (blue) and *Fmr1* KO (red) for each layer (I-VI). B: Spike rate and C: Fano factor were calculated over 2 s windows of spontaneous activity for 2 min. Each time window was compared with Student’s t-test and Cohen’s d effect size for both measures. p < 0.05 *, p < 0.01 **, p < 0.001 ***. Cohen’s d calculated for each t-test; S (small) d > 0.2, M (medium) d > 0.5, L (large) d > 0.8, VL (very large) d > 1.2, H (huge) d > 2. (For interpretation of the references to colour in this figure legend, the reader is referred to the web version of this article.)

Higher resting gamma may indicate higher or more variable spiking. We detected multi-unit spikes, plotting each layer’s average normalized PSTH over 2 s (50 trials) for *Fmr1* KO and WT mice in Fig. 2A. We calculated spike rate and Fano factor within each layer across subjects (Fig. 2B and C, respectively). For *Fmr1* KO mice, layer VI had a significantly higher Fano factor (*p* = 0.0081 / d = ’1.36) indicating increased variability of activity over time. There were no significant differences in spontaneous multiunit firing rates in any layer.

#### 3.1.1. CSD amplitude was reduced in the Fmr1 KO mice, compared to WT mice

**Fig. 3.**
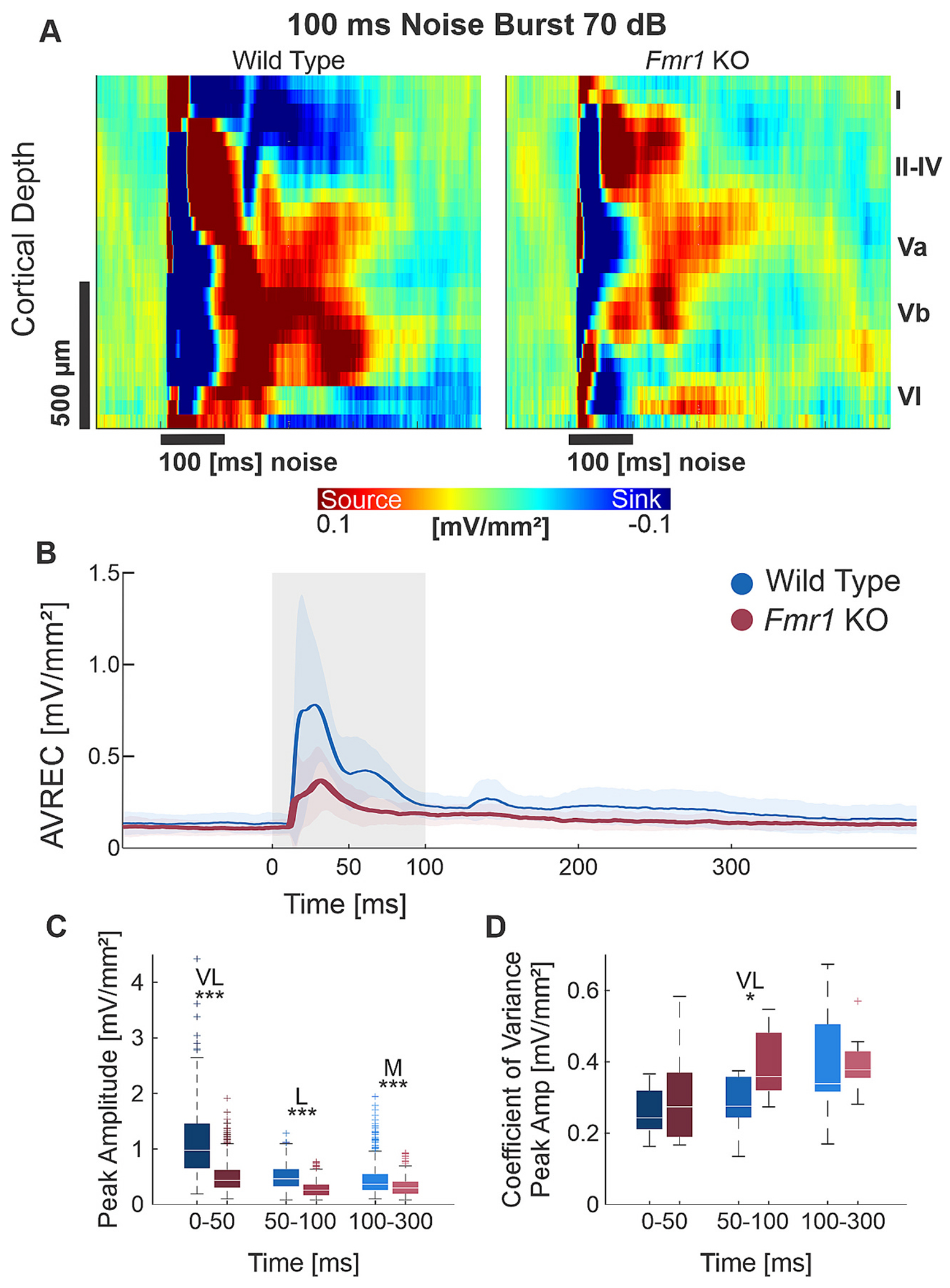
Reduced CSD amplitude in Fmr1 KO mice. A: Group-averaged CSD profiles of WT (left) and *Fmr1* KO (right) subjects, showing neural activity in response to 100 ms noise bursts at 70 dB SPL. Average layers are denoted on the right of each profile, though each subject has individual layer assignment. Current sinks (blue) indicate areas of population activity in cortical depth over time. B: Group-averaged average-rectified (AVREC) CSD traces for WT (blue) and Fmr1 KO (red) subjects of overall cortical column response to noise bursts. C: Traces were divided into 3 regions of interest: onset response (0–50 ms) and two post response windows (50–100 ms & 100–300 ms). Peak detection based on peak prominence was performed in each of these windows at a single-trial level. Peak amplitude as well as the coefficient of variance for peak amplitude (D), were compared by Student’s t-test with Bonferroni correction. p < 0.05 *, p < 0.01 **, p < 0.001 ***. Cohen’s d calculated for each t-test; S (small) d > 0.2, M (medium) d > 0.5, L (large) d > 0.8, VL (very large) d > 1.2. (For interpretation of the references to colour in this figure legend, the reader is referred to the web version of this article.)

CSD amplitude in response to 100 ms duration, 70 dB SPL noise bursts was reduced in the *Fmr1* KO mice, compared to WT mice (Fig. 3). We recorded LFPs in response to noise bursts at the same sound level (70 dB SPL) in both groups because there is no evidence for hearing loss (cochlear defects) in either the *Fmr1* KO mouse on the C57bl6/J genetic model (Chawla and McCullagh, 2022) or in humans with FXS (Roberts et al., 2005). No response threshold differences were seen in single unit recordings from WT and KO mice on the FVB background either (Rotschafer and Razak, 2013). To additionally ensure similar sensitivity in the mice used in the current study, we quantified the lowest sounds level at which a clear layer IV sink was elicited in the cortex in response to noise presented from 20 to 90 dB. In all WT mice, and in all but one Fmr1 KO mice, a response sink was visible at the lowest sound level tested (20 dB). In the remaining KO mouse, a sink was elicited at 30 dB SPL. Thus, there is not much of a difference in response thresholds justifying the use of the same sound level across genotypes.

Average layer designations are to the right of the *Fmr1* KO CSD profiles; layers are assigned per subject based on their cortical activation pattern in response to noise bursts for use in all analyses. The two major sinks correspond to inputs from the ventral division of the medial geniculate nucleus to A1, that mostly connect to two laminar bands corresponding to layer IV and the V/VI boundary (‘Vb’; Kimura et al., 2003; Romanski and LeDoux, 1993). The CSD was average rectified (AVREC) to produce an overall column trace of population activity, sink and source inclusive (Fig. 3B). Based on the average WT activity, three time windows were selected for peak detection: from 0 to 50 ms after sound onset, which includes initial thalamocortical inputs, and two longer latency activity windows between 50 and 100 ms and 100 and 200 ms after sound onset that may reflect corticocortical signaling (Kaur et al., 2004). *Fmr1* KO peak AVREC amplitude, based on peak prominence detection, was significantly lower for each time window (Fig. 3C, p = 1.85E-65 / d = 1.24, *p* = 8.25E-58 / d = 1.14, *p* = 8.01E-17 / d = 0.58, respectively). For each subject, a coefficient of variance (CV) was calculated on detected peak amplitudes to test the hypothesis that trial to trial response amplitude shows larger variability in the KO mice (Rotschafer and Razak, 2013; Bhaskaran et al., 2023). Fig. 3D shows that in a group comparison on the AVREC, variance was significantly higher in the post onset time window, 50 to 100 ms (*p* = 0.017 / d = ’1.21), in the *Fmr1* KO mice, but not during the other time windows. Taken together, these data indicate a reduction in population CSD amplitude within the local A1 column and an increase in inter-trial variability shortly after thalamic input (50–100 ms).

#### 3.1.2. Layer-specific abnormalities in the 40 Hz ASSR

**Fig. 4.**
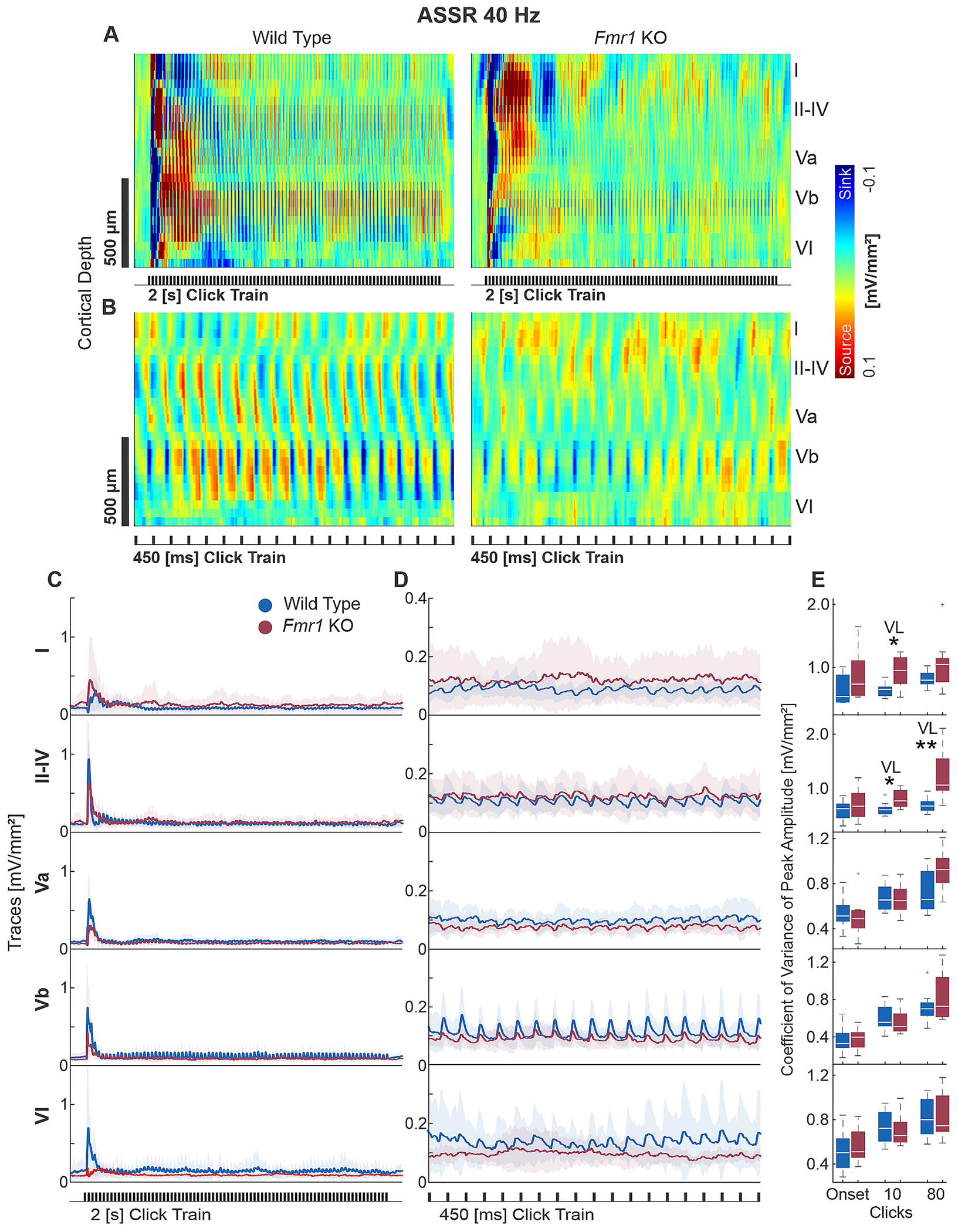
Reduced CSD following response to 40 Hz ASSR in Fmr1 KO mice. A. Group-averaged CSD profiles of WT (left) and *Fmr1* KO (right) subjects, showing neural activity in response to 2-s 40 Hz click trains. B: The same profiles showing just 450 ms of stimulus response (from 1150 to 1600 ms from onset). Average layers are denoted on the right. C: Sink activity traces for WT (blue) and Fmr1 KO (red) subjects from each layer, I, IV, Va, Vb, VI (top to bottom) in response to 2-s 40 Hz click trains. D: Sink activity of 450 ms of stimulus response (from 1150 to 1600 ms from onset). E: The coefficient of variance of the onset, the 10th, and the 80th click peak amplitudes calculated per subject compared across groups with Student’s t-test; p < 0.05 *, p < 0.01 **, p < 0.001 *** and Cohen’s d effect size; S (small) d > 0.2, M (medium) d > 0.5, L (large) d > 0.8, VL (very large) d > 1.2. (For interpretation of the references to colour in this figure legend, the reader is referred to the web version of this article.)

The 40 Hz auditory steady state response (ASSR) is a widely used stimulus paradigm to quantify temporal responses in the auditory cortex. There is reduced ASSR trial by trial consistency in *Fmr1* KO model rodents (Jonak et al., 2020; Kozono et al., 2020; Lovelace et al., 2020a), but whether layer-specific alterations underlie ASSR deficits in FXS remain unknown. Indeed, only one other study has examined laminar processing of ASSR in the auditory cortex in WT mice (Li et al., 2021). Fig. 4A shows the group-averaged CSD profiles of WT and *Fmr1* KO mice in response to 50 trials of 2-s 40 Hz click trains and Fig. 4B shows the ASSR of 450 ms of those click trains, excluding the onset response to the first click. There is a general reduction of CSD sinks in the *Fmr1* KO group. Sink activity was averaged across subject-specific layers, from IVI, for the full time window and the 450 ms window in Fig. 4C and D, respectively. In all layers except I, the *Fmr1* KO onset peak amplitude is significantly weaker (Table 2). CV of peak amplitude was calculated for the onset peak response as well as the 10th click, by the time the cortex habituated to the click train, and the 80th click, the last time-point of ASSR (Fig. 4E, Table 2). *Fmr1* KO layers I-IV had significantly higher variance in response peak amplitude across clicks. Variability was not significantly different in lower layers.

**Fig. 5.**
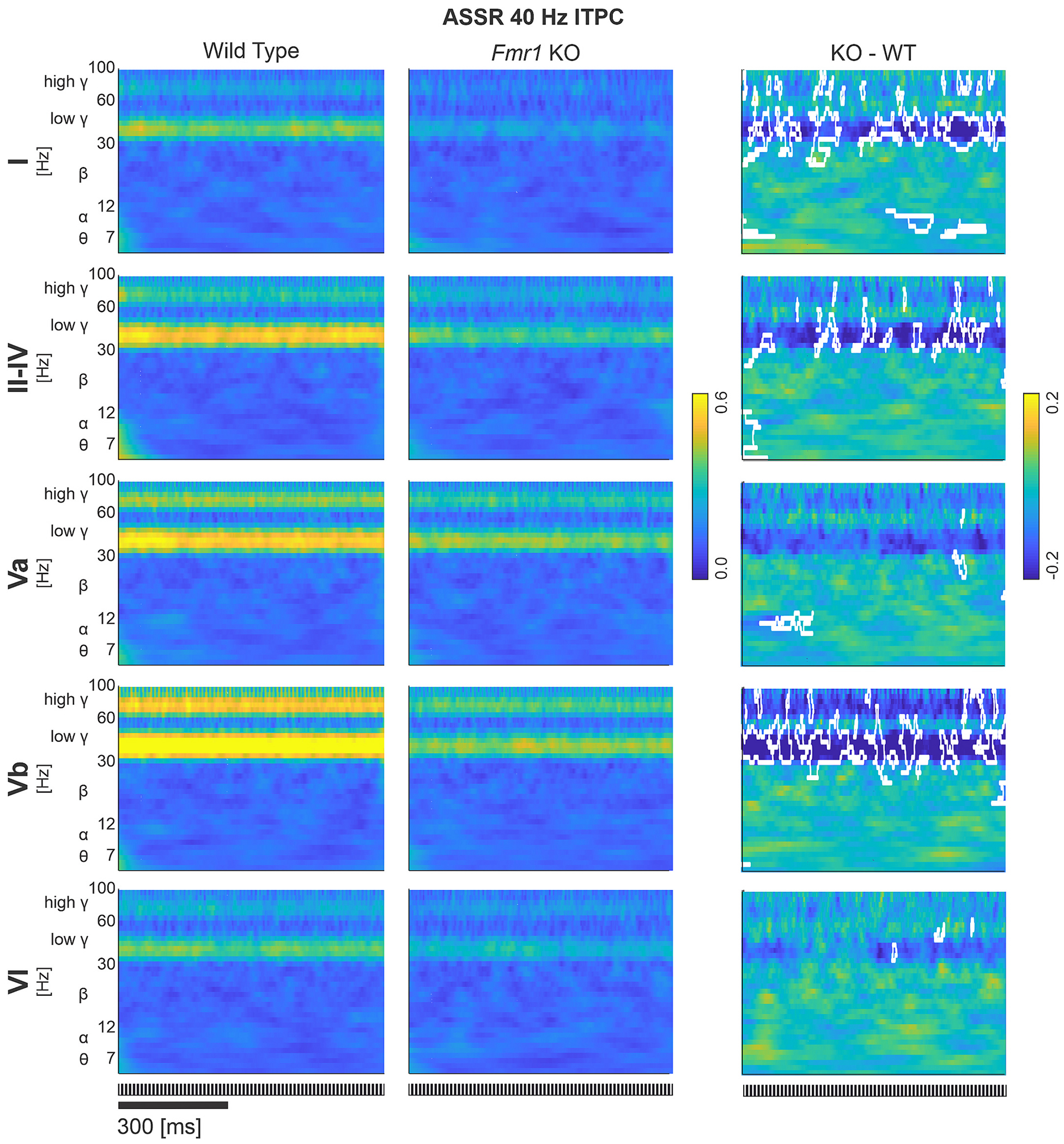
Laminar-specific reduction in 40 Hz ASSR ITPC in *Fmr1* KO mice. Group-averaged continuous-wavelet-transform-derived ITPC for WT (left) and Fmr1 KO (middle) subjects, and the difference between them (KO-WT, right) in response to 2-s 40 Hz click trains. Observed regions of significant difference, by point-wise Student’s t-test, are surrounded in white borders (determined by bwboundaries function in Matlab). Significance is verified by Permutation clustermass analysis with 1000 permutations and only boundaries over 3 pixels high and wide were kept.

Each subjec’s center layer channel was used to calculate continuous wavelet transformation, and the resulting time-frequency domain complex number output was transformed into intertrial phase coherence (ITPC) profiles per layer (I-VI). Fig. 5 shows the group-averaged ITPC for WT and *Fmr1* KO subjects, as well as the difference between them (KO-WT). Interestingly, in the WT mice, the maximum ITPC for 40 Hz ASSR appears in layer Vb, with relatively weaker ITPC in layers I and VI. There is an overall decrease in ITPC across layers in the *Fmr1* KO mice. Permutation clustermass analysis with the Matlab function bwboundaries was run on this group comparison, to determine areas where there was significant difference above a chance distribution. In layers I-IV, where *Fmr1* KOs had higher variability of peak amplitude response (Fig. 4E), there was significantly lower ITPC across the 40 Hz band. Layer Vb, where the WT group had the strongest ITPC at 40 Hz, there was also a significant reduction of ITPC in the *Fmr1* KO group. These data reveal layer-specific origins of ASSR inter-trial fidelity in WT mice and show a significant deficit in temporal processing in the *Fmr1* KO mice that seem to affect layers I and Vb the most.

### 3.2. Drastic abnormalities in gap-in-noise detection and processing in Fmr1 KO mice

**Fig. 6.**
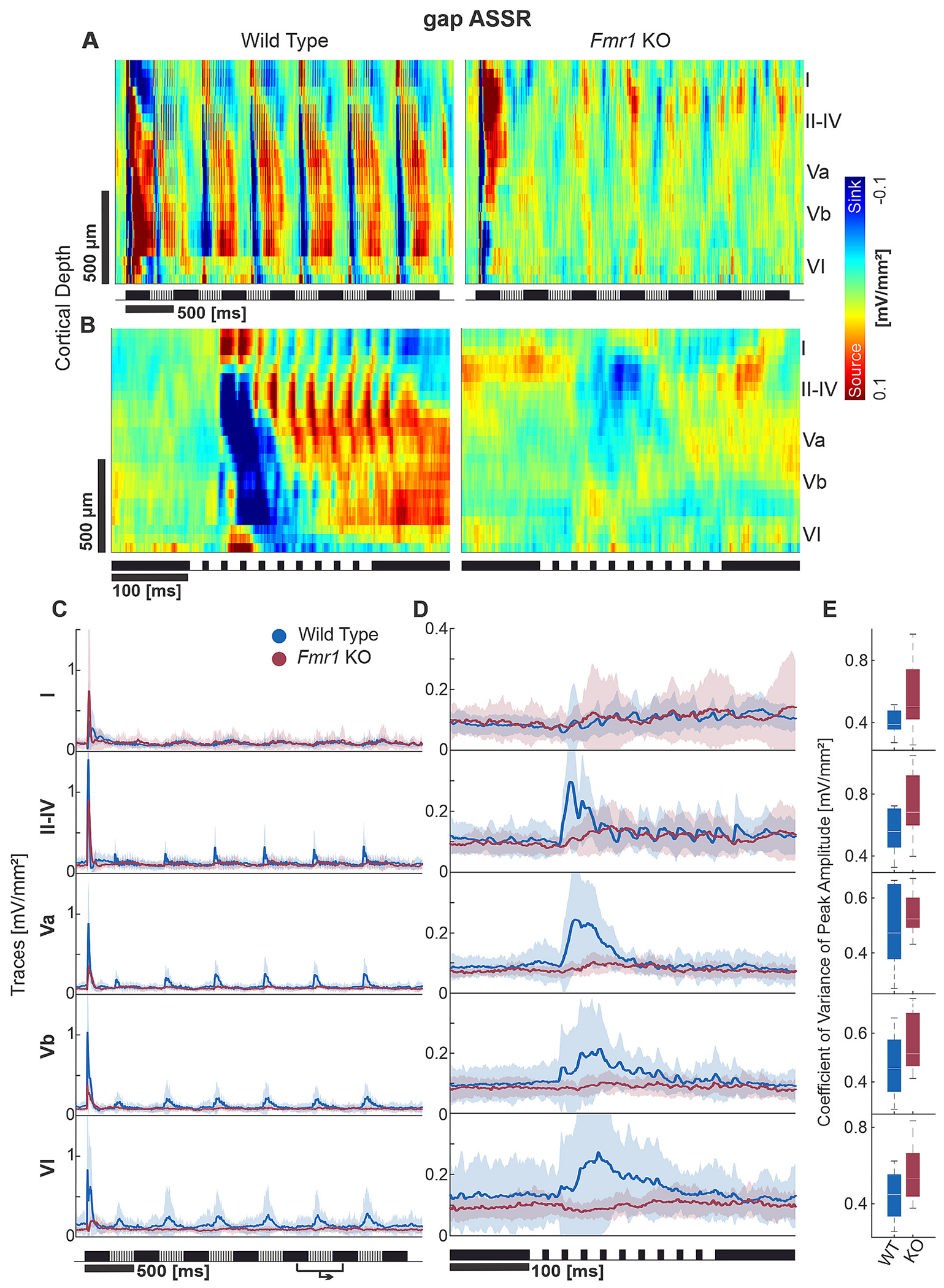
Greatly reduced CSD gap detection and following response in *Fmr1* KO mice. A: Group-averaged CSD profiles of WT (left) and Fmr1 KO (right) subjects, showing neural activity in response to ~4 s gap ASSR with 75 % modulation depth and 6 ms gaps in noise at 40 Hz. B: The same profiles showing just 450 ms of stimulus response (from 2150 to 2600 ms from onset). Average layers are denoted on the right. C: Sink activity traces for WT (blue) and Fmr1 KO (red) subjects from each layer, I, IV, Va, Vb, VI (top to bottom) in response to ~4 s gap ASSR with 75 % modulation depth and 10 ms gaps in noise at 40 Hz. D: Sink activity of 450 ms of stimulus response (from 2150 to 2600 ms from onset). E. The coefficient of variance of the peak amplitudes, detected from within the full 250 ms time window, calculated per subject compared across groups with Student’s t-test; p < 0.05 *, p < 0.01 **, p < 0.001 *** and Cohen’s d effect size; S (small) d > 0.2, M (medium) d > 0.5, L (large) d > 0.8, VL (very large) d > 1.2. (For interpretation of the references to colour in this figure legend, the reader is referred to the web version of this article.)

The 40 Hz gap-in-noise ASSR is a paradigm in which the gap widths can be modified systematically to quantify temporal processing in the auditory system (Rumschlag and Razak, 2021). Gap detection is used to measure temporal acuity (Phillips, 1999), an important feature of speech or vocalization perception. The laminar profile of cortical responses to short gaps in stimuli has not been studied previously. We used the laminar depth probe to record responses to short gaps embedded in noise in both WT and *Fmr1* KO mice. The stimulus consisted of alternating blocks of noise and gap-in-noise with varying gap widths at 75 % modulation depth (modulation depth was not changed in this study). Fig. 6A shows the group-averaged CSD profiles of WT and *Fmr1* KO mice in response to 50 trials of 6 ms gap ASSR and Fig. 6B shows activity across one gap-in-noise block. Supplemental Fig. 4 shows data for all gaps. There is a remarkable reduction in CSD sink activity across gap-in-noise blocks, despite similar onset responses (Fig. 6A) to clicks (Fig. 4A). This is further demonstrated with sink traces down the cortical layers for the full time window and the 450 ms window in Fig. 6C and D, respectively. Layers I-IV of the *Fmr1* KO group do show a comparatively small onset response to the gap-in-noise block (Fig. 6D), indicating some cortical recruitment to gap-in-noise blocks. The WT peak amplitude, detected from the full 250 ms gap-in-noise block time windows is significantly stronger down the layers, but with small effect size in I and II-IV and medium effect sizes in deeper layers (Table 2). The CV of these detected peak amplitudes of response was not significantly different, although the trend of *Fmr1* KO group’s higher variability in upper layers is still present (Fig. 6E, Table 2).

**Fig. 7.**
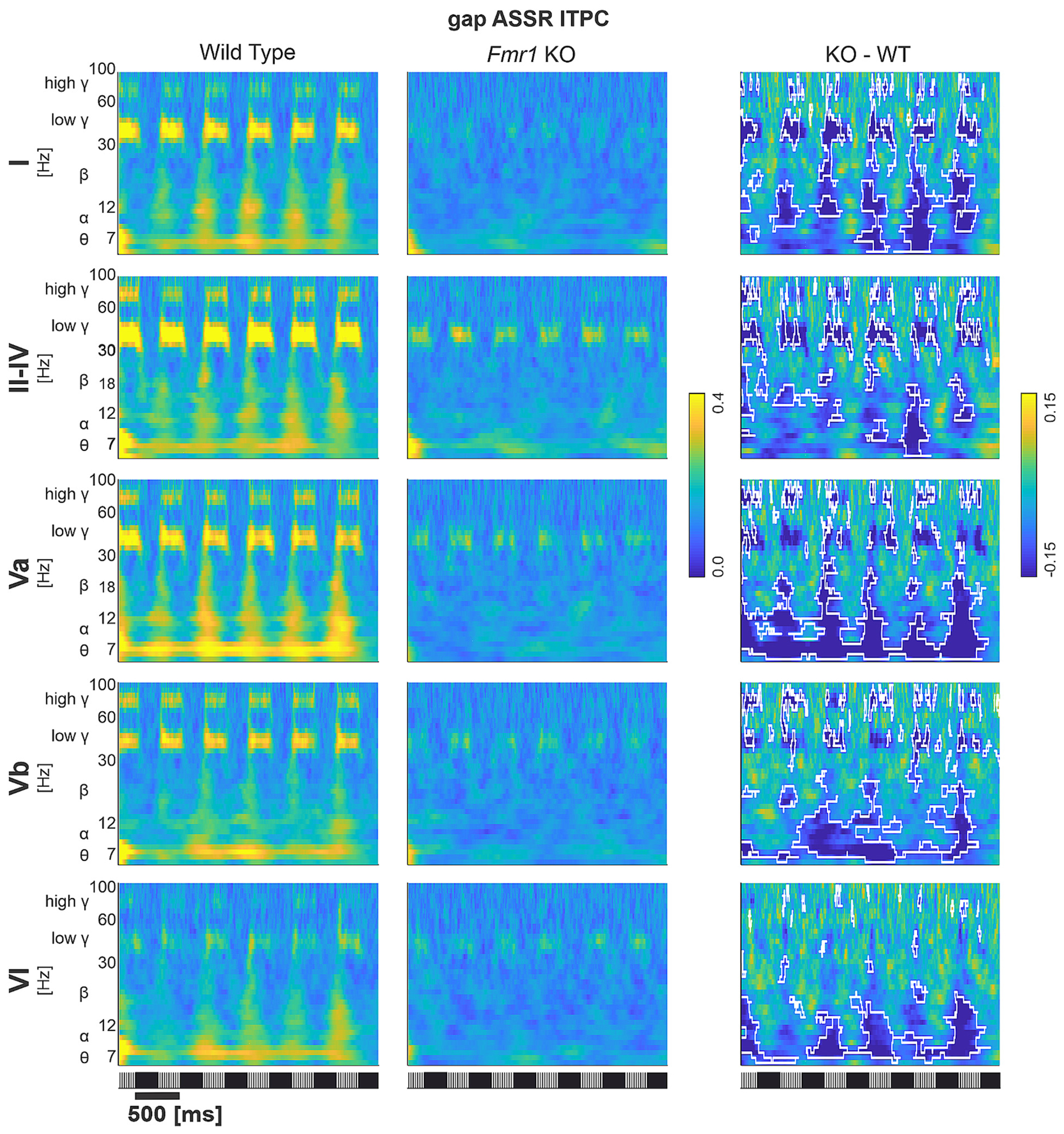
Greatly reduced gap-ASSR ITPC in *Fmr1* KO mice. Group-averaged continuous-wavelet-transform-derived ITPC for WT (left) and Fmr1 KO (middle) subjects, and the difference between them (KO-WT, right) in response to ~4 s gap ASSR with 75 % modulation depth and 6 ms gaps in noise at 40 Hz. Observed regions of significant difference, by point-wise Student’s t-test, are surrounded in white borders (determined by bwboundaries function in Matlab). Significance is verified by Permutation clustermass test with 1000 permutations and only boundaries over 3 pixels high and wide were kept.

ITPC profiles of each subjec’s center layer channels were calculated. Fig. 7 shows the group-averaged ITPC for WT and *Fmr1* KO mice, as well as the difference between them (KO-WT). With a dramatic reduction of ITPC down the layers, there are significant areas of difference, according to permutation clustermass analysis, for each gap-in-noise block at 40 Hz in every layer. In layer Va, the layer between granular and infra- granular thalamic input, there is a widely distributed theta/alpha band of activity throughout repetitive presentations of gap-in-noise blocks. This lower frequency band of activity is also significantly reduced in *Fmr1* KO mice. Taken together, these data reveal a reduced ability of the *Fmr1* KO mouse primary auditory cortex to detect and respond with high fidelity to short gaps in noise, a significant impediment to temporal processing.

### 3.3. Greater deep infragranular spike variability during ASSR

**Fig. 8.**
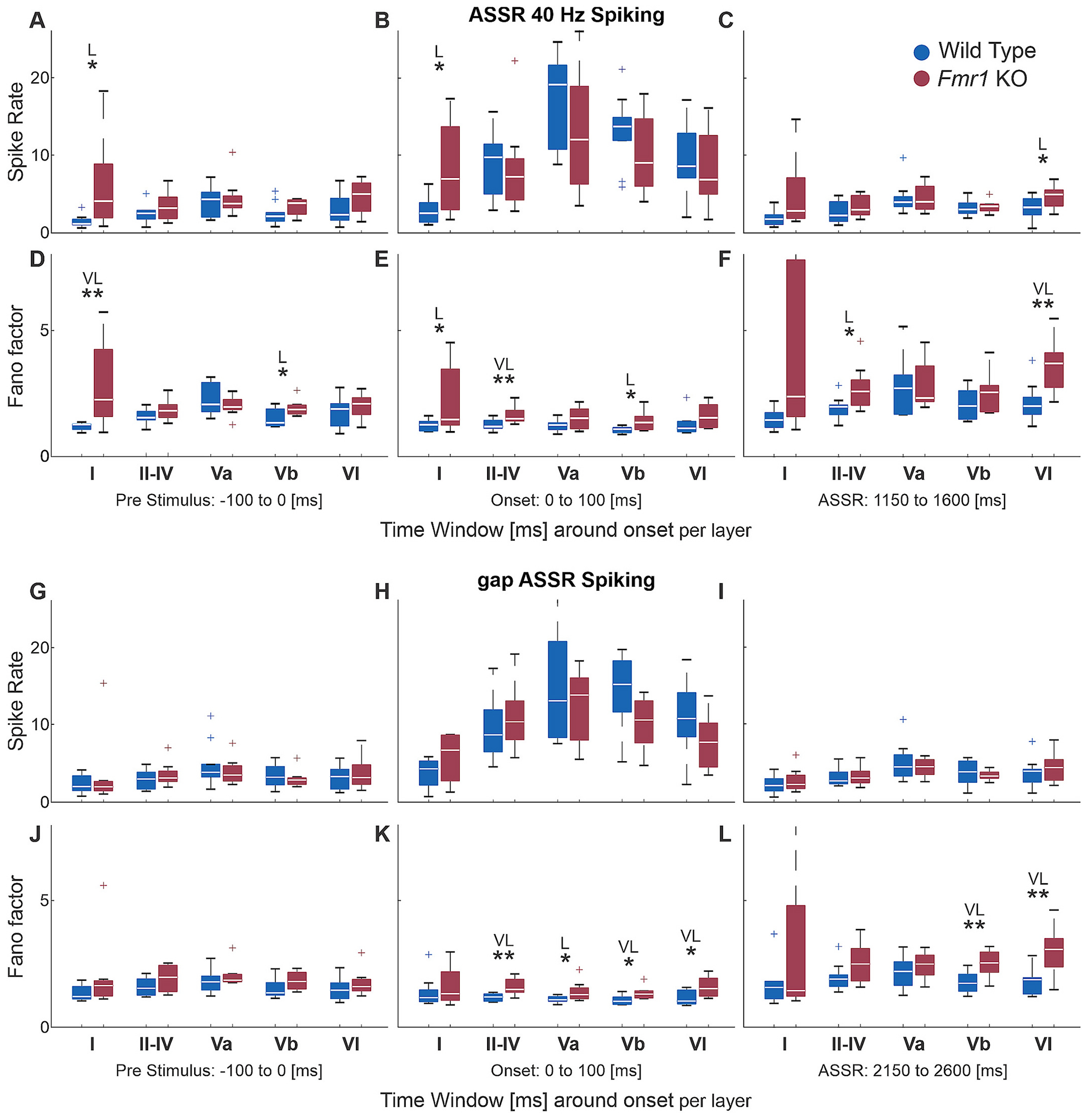
Higher ISI in layer VI during ASSR and broadly at noise or click onset in *Fmr1* KO mice. During 40 Hz ASSR, for each cortical layer (I-VI), spike rate was measured from 3 time windows: A: pre stimulus (−100 to 0 ms), B: onset (0 to 100 ms), and C: ASSR (1150 to 1600 ms – corresponding to Fig. 3D (manuscript)). Each layer time window was compared across groups, WT (blue) and *Fmr1* KO (red), with Student’s t and Cohen’s d effect size. Fano factor was calculated across layers for D: pre stimulus, E: onset, and F: ASSR time windows and compared across groups. During gap ASSR, for each cortical layer (I-VI), spike rate and Fano factor were measured from G/J: pre stimulus, H/K: onset, and I/L: ASSR (2150 to 2600 ms – corresponding to Fig. 5D (manuscript)). Student’s t and Cohen’s d were calculated for each comparison across groups. p < 0.05 *, p < 0.01 **, p < 0.001 ***. Cohen’s d calculated for each t-test; S (small) d > 0.2, M (medium) d > 0.5, L (large) d > 0.8, VL (very large) d > 1.2, H (huge) d > 2. (For interpretation of the references to colour in this figure legend, the reader is referred to the web version of this article.)

Multi-unit spikes were detected across both the click and gap ASSR stimuli and 3 time windows were selected as regions of interest for comparison: pre-stimulus (−100:0 ms), stimulus onset (0:100 ms), and based on the population activity, a time window where there is ASSR, also shown in Figs. 5D and 7D (clicks: 1150:1600 ms; gaps: 2150:2600 ms). In the *Fmr1* KO group pre-stimulus time-window, click ASSR at 40 Hz, shows an increase in spike rate (Fig. 8A, Table 3) in layer I and an increase in Fano factor (Fig. 8D, Table 3) in layers I and Vb. Clicks were presented in pseudorandomized trials at different presentation frequencies with 2 s inter-trial intervals, reducing the risk of temporal entrainment of cortical activity to repetitive stimuli. However, this prestimulus difference highlights a layer I spike rate and variability increase that is specific to the click ASSR stimulus. There are no differences between groups in the pre-stimulus window during gap ASSR presentation (Fig. 8G and J). Spike rate is significantly different for click ASSR in layer I during stimulus onset also, (Fig. 8B, Table 3) but is otherwise not different between groups during onset (Fig. 8B, H). Fano factor, however, varies across these time windows. In response to the 40 Hz click train onset (Fig. 8E, Table 3), Fano factor is significantly higher in layers I, II-IV, and Vb for *Fmr1* KO mice. In response to the noise onset for the gap ASSR stimulus *Fmr1* KO layers Va and Vb have significantly higher Fano factor. In the ASSR time-window for clicks, *Fmr1* KO mice had significantly higher spike rate in layer VI (Fig. 8C, Table 3), and Fano factor in layers II-IV and VI (Fig. 8F, Table 3). For gap ASSR, Fano factor was significantly higher in layer VI for the *Fmr1* KO group (Fig. 8L, Table 3). These data indicate that multiunit spike rates were similar for these stimuli across genotypes, but response variability was higher in the *Fmr1* KO mice in a layer specific manner.

## 4. Discussion

The major goal of the present study was to quantify layer-specific auditory cortical deficits using a preclinical model of global loss of FMRP. The global *Fmr1* KO mice was tested for translational relevance to the human FXS condition. A laminar analysis of WT and *Fmr1* KO mouse primary auditory cortex reveals major and novel layer-specific genotype differences in resting and sound driven responses. Analysis of resting (no stimuli) recordings show elevated power in low- (delta) and high- (gamma) frequency bands in *Fmr1* KO mice. Interestingly, the changes in different spectral bands appear in different layers, with layers I, V and VI showing the gamma band power elevation and layer II-IV showing the low-frequency power increase. Multiunit spike analysis reveals greater variability of spike rate over time in layer VI of the *Fmr1* KO mice, but no significant differences in spike rates across layers. The overall CSD amplitudes in response to noise bursts was significantly reduced in the *Fmr1* KO mice, particularly in the longer latency components. Dramatic laminar processing differences were observed in ITPC measurements in the ASSR paradigm. A strong reduction in ITPC in layers I-IV and Vb was seen the *Fmr1* KO mice compared to WT mice in 40 Hz ASSR. No major differences in 40 Hz ASSR ITPC were seen in layers Va and VI. For the gap-ASSR stimuli, ITPC was considerably reduced across all the layers in the *Fmr1* KO mice. CSD analysis revealed that the stimulus with gaps hardly registered a response in the *Fmr1* KO mice. Taken together, these data show major deficits in cortical population responses and highly abnormal responses to temporally modulated sounds in adult *Fmr1* KO mice compared to their WT counterparts. These data suggest novel laminar-specific mechanisms of abnormal auditory sensitivity and processing in FXS, that may link local cortical circuit deficits to macroscopic EEG response phenotypes in rodents and humans, and provide insights into how temporal processing deficits may lead to language deficits in ASD.

### 4.1. Resting gamma power and spontaneous activity in FXS

One of the most consistent phenotypes observed in FXS rodent models and humans with FXS is elevated resting EEG gamma power. In humans with FXS, gamma power correlates with social and sensory processing differences (Wang et al., 2017), and aperiodic gamma power correlates with better language abilities in developing boys (Wilkinson and Nelson, 2021). In mice, elevated gamma power is associated with abnormal habituation and working memory (Carlén et al., 2012)—traits that are associated with FXS. Consistent findings across rodent models and humans have led to suggestions that this phenotype may serve as a biomarker in FXS. Some (Jonak et al., 2020; Lovelace et al., 2018; Lovelace et al., 2020b), but not all (Kozono et al., 2020), studies have also reported elevated delta and/or theta resting EEG power in *Fmr1* KO rodents and in humans (Proteau-Lemieux et al., 2021; Smith et al., 2021; Van der Molen and Van der Molen, 2013; Wang et al., 2017). In males with FXS, higher theta was associated with improved speech-in-noise processing. Elevated theta and gamma power is also seen more widely in ASD, and in association with reduced alpha power, giving rise to an ‘U’ shaped elevation in spectral power changes (reviewed in Wang et al., 2013).

We observed both increased delta and gamma power in the *Fmr1* KO mice, but in different cortical layers. Layers I and V-VI carry most of the differences in gamma band, while layer II-IV show low frequency differences. The EEG signals that demonstrate elevated gamma and low frequency power may, therefore, reflect the summed patterns of activity of signaling from pyramidal neurons from layers I/V-VI and II-IV, respectively, leading to the ‘U’ shaped elevation seen in FXS and ASD. These data point to specific hypotheses regarding underlying mechanisms. Broadband gamma power elevation may reflect abnormal activation of NMDA receptors in parvalbumin (PV) positive neurons in deep cortical layers. Deficient PV neuron activation causes elevated broadband gamma power and leads to specific sensory processing deficits (Carlén et al., 2012; Guyon et al., 2021), including abnormal gap detection (Keller et al., 2018), as seen with the gap-ASSR paradigm. Asynchronous and increased spontaneous activity will also elevate broadband gamma power (Ray and Maunsell, 2011). We have previously shown abnormal development of perineuronal nets (PNNs) around PV neurons in the auditory cortex of *Fmr1* KO mice (Wen et al., 2018), which in turn may result from elevated levels of matrix metalloproteinase-9 (MMP9) in the *Fmr1* KO mice. In the visual cortex, loss of PNNs leads to elevated gamma band power (Lensjø et al., 2017). Staining in these studies reveal that while PV neurons are distributed throughout the layers in the A1, PNN density is highest in layer IV and in infragranular layers. Layers I-III had relatively less PNN density. Measurement of multi-unit spike activity associated with these layer showed that layer I had a trending increase in spike rate and variability (Fano factor). Spiking in layer VI of *Fmr1* KO mice was significantly more variable. Infragranular pyramidal neurons send afferents up the cortical column to layer I for wider cortical spread of columnar activity. Layer I is largely made up of dendrites and axons connecting the local column to near and distal areas of the neocortex. Therefore, higher spiking activity in infragranular layers may translate to increased gamma and subthreshold population activity in layer I. Our previous single unit recordings from the A1 also showed increased response variability, but layer-specific differences were not analyzed (Rotschafer and Razak, 2013). These data are consistent with findings in the somatosensory cortex in which *Fmr1* KO mice show increased paw tactile response variability, likely due to endogenous noise sources such instability of membrane potentials (Bhaskaran et al., 2023). However, these recordings were limited to layers II-III, and it is not known if the layers with most variability in the auditory cortex also show elevated noise in the somatosensory cortex. An emerging theme in cortical responses in *Fmr1* KO mice, therefore, is increased response variability with some limited evidence for increased spiking. This may relate to a general finding in ASD regarding increased response variability (David et al., 2016; Robertson and Baron-Cohen, 2017). If there is a causal association between MMP-9 and PNN deficits and the observed layer-specific gamma deficits is unclear and future studies will examine *Fmr1* KO mice in which MMP-9 is also reduced (Wen et al., 2018) and with specific MMP9 inhibitors (Pirbhoy et al., 2020).

### 4.2. Sound driven responses show layer-specific genotype differences

The CSD profile in both genotypes showed normal layer-specific sink/source activity in response to noise bursts. This includes the two short latency sinks corresponding to layer IV and the V/VI boundary (Vb; Kimura et al., 2003; Romanski and LeDoux, 1993) that receive thalamocortical inputs, and longer latency sinks that correspond to corticocortical connectivity in the superficial layers (Kaur et al., 2004). AVREC peak amplitudes in response to noise bursts were significantly smaller in *Fmr1* KO mice compared to the WT mice for short, medium, and long latency responses. Despite differential cortical activity to gap and 40 Hz ASSR conditions, phenotype differences in onset responses were consistent across stimuli. The medium latency AVREC (50 to 100 ms after noise onset) also showed significantly more variability from trial to trial. Variability was consistent across groups within the time window of direct thalamocortical recruitment (0 to 50 ms) and therefore became more variable within the A1 directly after onset. This suggests that at least some of the higher trial-to-trial variability, which contributes to lower ITPCs, originates in the cortex.

Both 40 Hz click trains and gap-in-noise ASSR were used to study trial-to-trial variability of temporal processing across layers. ASSR stimulus paradigms are used to measure the ability of the EEG generators to synchronize consistently to time varying stimuli and provide a valuable tool to probe temporal processing and underlying excitatory/ inhibitory circuits. Temporal processing is critical for speech processing in humans (Shannon et al., 1995; Tallal et al., 1998), and rodent studies of ASSR may provide a bridge to understanding circuit deficits leading to speech/language impairments. The 40 Hz ASSR deficit in schizophrenia and ASD has been suggested as an endophenotype to study circuit pathology in these neurodevelopmental disorders (Tada et al., 2020). Previous studies have shown significant deficits in ITPC for ASSR and gap-ASSR in the *Fmr1* KO mice and in humans with FXS (Croom et al., 2023; Ethridge et al., 2017; Jonak et al., 2020). In Croom et al. (2023), the gap-ASSR paradigm was investigated via EEG recordings in *Fmr1* KO and WT mice on the FVB background strain during development (ages p21, p30 and p60). While there was significant differences in gap-ASSR ITPC in p21 and p30, no significant genotype differences were seen in the p60 mice. However, deficits are seen in adult C57bl6 strain in the present study that may reflect strain differences in development of gap-ASSR and/or differences in spatial resolution of EEG vs CSD measured outcomes.

To identify potential layer-specific mechanisms of *Fmr1* KO deficits in ASSR conditions, we used the depth probe to record click ASSR and gap ASSR at 40 Hz. Only one study has previously tested if 40 Hz ASSR has layer-specific mechanisms and concluded that the thalamocortical input, ~750 μm deep (labeled layer IV in their manuscript, but more consistent in CSD literature to be the border of V and VI) contributes most to the power and consistency of the 40 Hz ASSR (Li et al., 2021). Our findings are consistent with this conclusion with layer Vb showing the strongest ASSR ITPC in the WT mice, followed by layer II-IV. Remarkably reduced ITPC was observed across all layers in the *Fmr1* KO mice, with significant differences emerging in layers I, II-IV and Vb. No difference was seen in layers Va and VI, indicating that EEG measures of ASSR deficits in FXS arises through laminar differences in EEG generators. Recent work in humans indicate that GABAa receptor signaling, probably through activation of NMDA receptors on PV+ GABAergic neurons, shapes the 40 Hz ASSR (Toso et al., 2024). Basal forebrain PV/GABA neuron projections enhance cortical 40 Hz ASSR (T. Kim et al., 2015). The abnormal ASSR ITPC seen in the *Fmr1* KO mouse cortex may arise from reduced NMDA expression in PV neurons, abnormal long distance basal forebrain to A1 projections (Z. Zhang et al., 2021) and/or abnormal GABA receptors in the cortex (Braat et al., 2015). Future studies will address these mechanisms.

In contrast to 40 Hz ASSR, the granular thalamic input layer IV produces the strongest gap-ASSR ITPC in WT mice. This suggests potentially different mechanisms for the generation of these two types of steady state responses. The gap detection and following response may arise in subcortical sites and inherited in the A1 as suggested by deficits in the input thalamic layers. A theta rhythm is also seen in gap-ASSR responses in the WT, even though the periodicity of the stimulus was at 40 Hz every 250 ms. This may reflect onset responses to each gap-in-noise block of the stimulus train. The *Fmr1* KO brain does not show much trial-to-trial consistency in gap-ASSR, with ITPCs hardly emerging beyond the background. Therefore, significant differences were seen in multiple layers, with layer Va and VI showing differences for the theta band response. The identified disruptions in detecting and responding to short temporal gaps in auditory stimuli may underlie speech/language disruptions in FXS and ASD, more broadly (Cabrera and Gervain, 2020; Shannon et al., 1995; Tallal et al., 1998; Trehub and Henderson, 1996). Notably, impaired gap detection thresholds in children with autism were associated with lower phonological processing scores (Foss-Feig et al., 2017). The *Fmr1* KO mouse data points to significant difficulty in cortical neural generators, and underlying gap detection circuits (Keller et al., 2018; Weible et al., 2014), to detect and respond to short gaps, suggesting a path forward to understanding circuit pathology and pre-clinical therapeutic approaches specifically targeting speech/language deficits in FXS and ASD.

### 4.3. Higher spike rate variability differential across stimuli and layers

Spike rate and variability were calculated from multi-unit spiking data in pre-stimulus, onset, and ASSR time windows. Spike rates are significantly different during click ASSR in the pre-stimulus and onset windows in layer I, and during ASSR in layer VI but are otherwise equivalent. Spike rate variability, measured as Fano factor, is significantly different across time windows and layers in response to clicks. Surprisingly, gap ASSR elicits equivalent spike rates between genotypes, despite having greater reduction of population activity and ITPC. Spike rate variability is significantly different in layer V during onset and in Layer VI during ASSR. During the ASSR spike detection window, both stimuli produce greater spike variability in *Fmr1* KO mice in layer IV, in line also with greater variability in this layer during spontaneous activity.

The reduced CSD response amplitudes and increased or similar spike rates in FXS raises an interesting observation about cortical processing in FXS. If LFPs, as subthreshold input, are reduced, but neuronal output, or spiking, is similar or elevated, there may be a mechanism, either intrinsic to neurons or from a circuit perspective, that produces abnormal cortical gain in *Fmr1* KO auditory cortex. The elevated power and spiking rate may be a compensatory response to reduced inputs across cortical layers. In *Fmr1* KO mice, any reduction in inputs is likely to be locally in the cortex, as the inferior colliculus shows enhanced activity during early development (Nguyen et al., 2020), and auditory brainstem responses do not show any major hearing loss in *Fmr1* KO mice compared to WT mice (Chawla and McCullagh, 2022). Alternately, reduced synchrony of field potentials, likely due to increased variability, may lead to lower LFP/CSD amplitudes in *Fmr1* KO mice. Disambiguating these possibilities across development will be critical in understanding sensory dysfunction in FXS. Taken together, we have replicated the *Fmr1* KO phenotypes of increased low and high frequency resting oscillation power, reduced phase locking, and higher variability using a higher spatial resolution recording method (CSD, compared to EEGs), suggesting local deficits in the primary auditory cortex. The major suggestion of the present data is that cortical hyperexcitability may appear in response to reduced cortical layer inputs. Future studies will record LFP/CSD in *Fmr1* KO mice in early development (at hearing onset ~12 days after birth). To disentangle the micro- and mesoscopic scale of cortical microcircuitry in FXS mouse models, future studies will also analyze spike – LFP coherence and coherence between layers.

### 4.4. Methodological considerations

The results show novel layer-specific changes in auditory processing in the *Fmr1* KO mice, including significant decline in temporal processing. It is important to note that these data were collected in this study under urethane/xylazine anesthesia. Hays et al. (2011) showed that UP states, which are spontaneously occurring persistent activity states, were elevated in duration in the *Fmr1* KO mouse somatosensory cortical slices. A similar increase was also seen in vivo in urethane-anesthetized *Fmr1* KO mice, indicating that a commonly reported cortical circuit phenotype is replicated in vivo under urethane anesthesia. Auditory hyperexcitability seen in awake mouse EEG responses of *Fmr1* KO mice are also seen in urethane-anesthetized mouse recordings (Rotschafer and Razak, 2013; Wen et al., 2018). Likewise, urethane-anesthetized *Fmr1* KO mice showed hyperexcitability to tactile whisker stimulation as measured with voltage sensitive dye imaging (Zhang et al., 2014). Such phenotype replicability across different conditions and stimuli, in a manner analogous to FXS human responses, may be due to the mechanisms of action of urethane compared to other anesthetics.

The commonly used gas anesthetic, isoflurane, reduces peripheral auditory function (Ruebhausen et al., 2012) and tone-responsive cortical neurons (Noda and Takahashi, 2015). Compared to other injectable anesthetics such as pentobarbital which suppresses neural activity (Wan and Puil, 2002) or ketamine which causes cortical layer specific effects (Deane et al., 2020), auditory cortical responses are relatively well preserved under urethane (Maggi and Meli, 1986). Anesthetic doses of urethane increase the response of auditory neurons to tones by lowering thresholds, enhancing evoked spiking, and broadening frequency tuning (Huang et al., 2022). Huang et al. attributed this to a possible increase in the ratio of excitatory/inhibitory inputs to neurons. Therefore, the present data were likely not affected by suppressed cortical responses. Critical for the current study is that WT and *Fmr1* KO mice responded similarly to the anesthetic dose, and were not different in the dose required or the length or strength (as measured with the toe pinch response) of anesthesia. However, whether the brain responds differently to urethane in WT versus *Fmr1* KO mice remains unknown. Anesthesia, while providing the advantage of stable responses which may be suitable for basic sensory processing of task-irrelevant stimuli, alters cortical dynamics which may not fully reflect the fully awake, behaving state relevant to sensory hypersensitivity in FXS. Future work will perform studies in awake *Fmr1* KO and WT mice to understand if awake versus anesthetic responses differ in the *Fmr1* KO mice.

A second important methodological issues is that we performed spiking rate and variability analysis based on multi-unit spiking data and relatively simple acoustic stimuli. Our focus was on the LFP recording to gain insight on layer-specific contributions to similar EEG phenotypes in humans with FXS and the *Fmr1* KO rodent models. We have used simple auditory stimuli to address cortical layer specificity to align with previous studies in both humans and rodents (reviewed in Razak et al., 2021). Future studies should examine the relationship between responses to stimuli such as ASSR and gap-ASSR, and more complex species-typical vocalizations in both mice and humans to understand the relationship between ASSR type responses and language function in ASD. Moreover, the trade-off with electrode impedance for LFP recordings is a reduction in the quality of single-unit spike detection. This study used the z-coated probes (Neuronexus), which are intended to provide good spike detection for multiunit analysis. Additional analysis such as spike timing patterns and reliability for complex vocalizations would benefit from spike sorting and single unit analysis (Huetz et al., 2009; Schnupp et al., 2006).

### 4.5. Source of cortical deficits

While we report significant and novel layer-specific deficits in cortical responses, the data presented in this study cannot address where the deficits originate in the auditory pathway. FMRP is expressed across the auditory system (Zorio et al., 2017), and the deficits observed in cortical recordings may have their sources at multiple levels of the auditory pathway (Brown and Harrison, 2010; McCullagh et al., 2020). Given that the *Fmr1* KO mouse also shows auditory critical period plasticity deficits (Kim et al., 2013), the adult mouse recordings may reflect deficits and homeostatic compensatory mechanisms accrued over development (Antoine et al., 2019; Bülow et al., 2019). The literature provides a few clues on possible sources of auditory cortical deficits. First, the auditory deficits observed in the *Fmr1* KO mice are likely due to changes in central auditory processing, and not due to cochlear deficits. Peripheral hearing, at least in the low- to mid-frequency hearing range appear normal in *Fmr1* KO mice on the same genetic background as the present study (Chawla and McCullagh, 2022). In the adult C57bl6 mice, there is no significant threshold difference in hearing at any frequency tested. There is a trend for a non-significant increase in threshold at the highest frequencies tested (>32 kHz). This is unlikely to affect our results as we used noise and clicks that have energy in the lower frequencies of the mouse audible range where there was no threshold difference or ABR wave I amplitude differences.

To understand mechanisms of cortical hyperexcitability and temporal processing deficits at the systems level, a number of specific Cre lines can be used in the future. In addition to the auditory cortex, previous studies have reported abnormal responses across a number of brainstem and midbrain regions of the auditory pathway in global *Fmr1* KO mice. This includes hyper-responsiveness in the inferior colliculus (Nguyen et al., 2020; Sibille et al., 2022) and lateral superior olive (Garcia-Pino et al., 2017), and abnormal plasticity in the medial nucleus of the trapezoid body and cochlear nucleus (Strumbos et al., 2010). When FMRP is deleted only from the forebrain excitatory neurons using the Nex1-cre and CamKII-cre lines, sub-cortical sites show normal FMRP expression (Lovelace et al., 2020b). In these mice, a number of the same deficits seen in global *Fmr1* KO mice are present. This includes high gamma power, abnormal auditory steady state responses and hyperactivity. This suggests at least part of the cortical deficits have a local cortical circuit origin. When FMRP is deleted only in brainstem and midbrain using the Ntsr1-cre line, temporal processing deficits are present, but not the high gamma or single trial power seen in global *Fmr1* KO mice (Holley et al., 2022). These data appear to suggest that the layer-specific power deficits seen in the present study may originate from cortical deficits, perhaps linked to function of parvalbumin neurons and perineuronal nets. Whereas the strong layer-specific temporal processing deficits may have a sub-cortical origin. It is necessary for future depth electrode studies to record from these Cre-lined based *Fmr1* KO mice.

## Supplementary Material

1

## Figures and Tables

**Table 1:** Corresponding to Fig. 1, *p* value results after Bonferroni correction, F, and Cohen’s d effect size results for frequency bands delta, theta, alpha, beta, low and high gamma, and for pooled bands. Significant results, *p* < 0.05, and effect sizes above medium, d < 0.5, are in bold.

	Delta			Theta			Alpha			Beta		
Layer	p	F	d	p	F	d	p	F	d	p	F	d

I	7.27	4.70	−0.07	23.59	1.25	0.02	1.68	0.72	0.11	**1.92E-03**	0.48	0.24
II-IV	**2.83E-40**	3.94	**−0.84**	**8.11E-06**	1.97	−0.31	**1.23E-03**	0.55	0.24	**0.02**	0.70	0.21
Va	15.04	1.57	−0.04	4.40	0.97	0.09	17.24	1.10	0.03	**5.37E-08**	2.44	−0.36
Vb	28.73	1.29	0.00	9.74	1.07	−0.06	**6.34E-06**	2.10	−0.31	**6.31E-03**	0.95	−0.22
VI	0.15	1.60	0.17	2.15	1.79	−0.11	**7.32E-08**	2.44	−0.36	**3.30E-10**	1.12	−0.41
	Low Gamma			High Gamma			Pooled					
	p	F	d	p	F	d	p	F	d			
I	**1.37E-04**	1.44	−0.27	**2.78E-35**	3.44	**−0.78**	**1.35E-16**	2.08	**−0.52**			
II-IV	4.16	0.76	0.09	1.88	0.91	−0.11	16.80	0.83	−0.03			
Va	**8.96E-06**	1.78	−0.31	**1.90E-06**	1.63	−0.32	**2.17E-07**	2.00	−0.34			
Vb	0.06	0.54	−0.18	**5.38E-06**	0.80	−0.31	**4.96E-05**	0.74	−0.29			
VI	**8.87E-31**	1.60	**−0.72**	**8.78E-25**	2.34	**−0.64**	**4.24E-31**	1.88	**−0.72**			

**Table 2:** Corresponding to Fig. 4 and Fig. 6 *p* values after Bonferroni correction, F, and Cohen’s d effect size results for peak amplitude (top) and peak amplitude CV (bottom) for 40 Hz ASSR onset, 10th, and 80th clicks, and for gap ASSR block. Significant results, p < 0.05, and effect sizes above medium, d < 0.5, are in bold.

	40 Hz ASSR									Gap ASSR		
	Onset Peak Amp			10th Click Peak Amp			80th Click Peak Amp			Gap-in-Noise Peak Amp		
Layer	p	F	d	p	F	d	p	F	d	p	F	d

I	15.00	0.28	−0.25	**0.04**	0.61	−0.18	**4.67E-04**	0.23	−0.27	**2.29E-07**	0.49	−0.20
II-IV	**7.83E-11**	0.76	0.45	10.04	1.07	0.03	**7.20E-03**	0.32	−0.21	**2.67E-27**	0.93	0.43
Va	**8.23E-27**	1.97	**0.74**	14.97	1.94	0.20	14.43	1.09	0.12	**1.25E-79**	2.60	**0.78**
Vb	**1.23E-23**	1.21	**0.69**	15.00	2.76	0.28	14.64	1.30	0.13	**6.72E-66**	2.58	**0.71**
VI	**5.15E-29**	2.82	**0.79**	15.00	1.84	0.30	15.00	1.44	0.25	**5.26E-44**	1.96	**0.58**
	Onset Peak Amp CV			10th Click Peak Amp CV			80th Click Peak Amp CV			Gap-in-Noise Peak Amp CV		
	p	F	d	p	F	d	p	F	d	p	F	d
I	0.74	4.88	**−0.69**	**0.03**	12.88	**−1.44**	0.47	6.52	**−0.79**	0.24	5.94	**−0.96**
II-IV	1.45	6.56	**−0.50**	**0.04**	25.49	**−1.34**	**7.93E-03**	7.59	**−1.68**	0.28	11.38	**−0.95**
Va	3.19	8.58	0.22	4.79	25.29	0.02	0.20	27.01	**−1.02**	1.56	49.07	**−0.51**
Vb	4.47	10.65	−0.06	2.10	18.07	0.38	1.15	11.84	**−0.57**	0.90	20.00	**−0.67**
VI	2.71	14.78	−0.29	3.41	26.43	0.19	4.29	16.02	−0.08	0.45	12.19	**−0.84**

**Table 3:** Corresponding to Fig. 8, p values after Bonferroni correction, F, and Cohen’s d effect size results for spike rate and Fano factor in 3 time windows (pre, onset, ASSR) for 40 Hz ASSR onset (top) for gap ASSR (bottom). Significant results, p < 0.05, and effect sizes above medium, d < 0.5, are in bold.

	40 Hz ASSR								
	Spike Rate Pre-Onset			Spike Rate Onset			Spike Rate ASSR		
Layer	p	F	d	p	F	d	p	F	d

I	**0.02**	1.04	**−1.10**	**0.04**	1.09	**−1.02**	0.05	1.05	**−0.92**
II-IV	0.20	3.17	**−0.61**	0.73	1.85	0.16	0.23	6.50	**−0.57**
Va	0.66	3.46	−0.20	0.20	2.58	**0.61**	1.00	5.94	0.00
Vb	0.13	10.83	**−0.73**	0.15	4.09	**0.69**	0.77	22.50	−0.14
VI	0.09	5.05	**−0.84**	0.56	3.33	0.27	**0.04**	10.94	**−1.04**
	Fano Factor Pre-Onset			Fano Factor Onset			Fano Factor ASSR		
	p	F	d	p	F	d	p	F	d
I	0.05	2.65	−1.33	**0.04**	2.80	**−1.01**	0.06	0.78	**−0.91**
II-IV	0.14	19.65	**−0.70**	**0.01**	18.19	**−1.34**	**0.03**	8.96	**−1.08**
Va	0.27	28.08	**0.54**	0.06	12.05	**−0.89**	0.96	9.61	−0.02
Vb	**0.02**	40.09	**−1.14**	**0.03**	14.57	**−1.19**	0.16	11.41	**−0.67**
VI	0.37	16.44	−0.43	0.06	12.75	**−0.91**	**2.54E-03**	12.07	**−1.61**
	Gap ASSR								
	Spike Rate Pre-Onset			Spike Rate Onset			Spike Rate ASSR		
	p	F	d	p	F	d	p	F	d
I	0.45	0.56	−0.34	0.14	0.90	**−0.69**	0.37	3.26	−0.43
II-IV	0.44	5.01	−0.37	0.43	6.90	−0.38	0.78	7.59	−0.13
Va	0.45	5.14	0.38	0.38	6.94	0.44	0.51	15.21	0.33
Vb	0.70	6.96	0.19	0.07	9.13	**0.96**	0.54	31.09	0.31
VI	0.51	3.24	−0.31	0.13	4.79	**0.77**	0.52	5.26	−0.31
	Fano Factor Pre-Onset			Fano Factor Onset			Fano Factor ASSR		
	p	F	d	p	F	d	p	F	d
I	0.22	1.86	**−0.61**	0.47	4.78	−0.36	0.66	1.27	−0.22
II-IV	0.14	13.80	**−0.76**	**3.13E-03**	21.45	**−1.58**	0.09	10.12	**−0.84**
Va	0.39	20.19	−0.42	**0.03**	12.24	**−1.04**	0.35	22.34	−0.46
Vb	0.08	26.64	**−0.90**	**0.02**	29.14	**−1.26**	**3.55E-03**	22.91	**−1.60**
VI	0.29	10.91	**−0.51**	**0.02**	15.28	**−1.24**	**5.95E-03**	10.15	**−1.46**

## Data Availability

Data will be made available on request.

## Appendix A. Supplementary data

Supplementary data to this article can be found online at https://doi.org/10.1016/j.nbd.2025.106963.

## Glossary

ASD: Autism Spectrum Disorders
ASSR: auditory steady state response
AVREC: average rectified CSD
CSD: current source density
EEG: electroencephalography
Fmr1 KO: mouse model for FXS syndrome – Fmr1 gene knock out
FMRP: Fragile X Messenger Ribonucleoprotein
FXS: Fragile X Syndrome
ITPC: inter trial phase coherence
LFP: local field potential
WT: wildtype
