# Supplementary material for: Cortical layer-specific abnormalities in auditory responses in a mouse model of Fragile X Syndrome
Source: Neurobiol Dis. Author manuscript; Available in PMC 2026 Jun 11. (PMC13255874; doi:10.1016/j.nbd.2025.106963)
Supplement: 1 [file NIHMS2176224-supplement-1.docx]

## Supplemental Figures


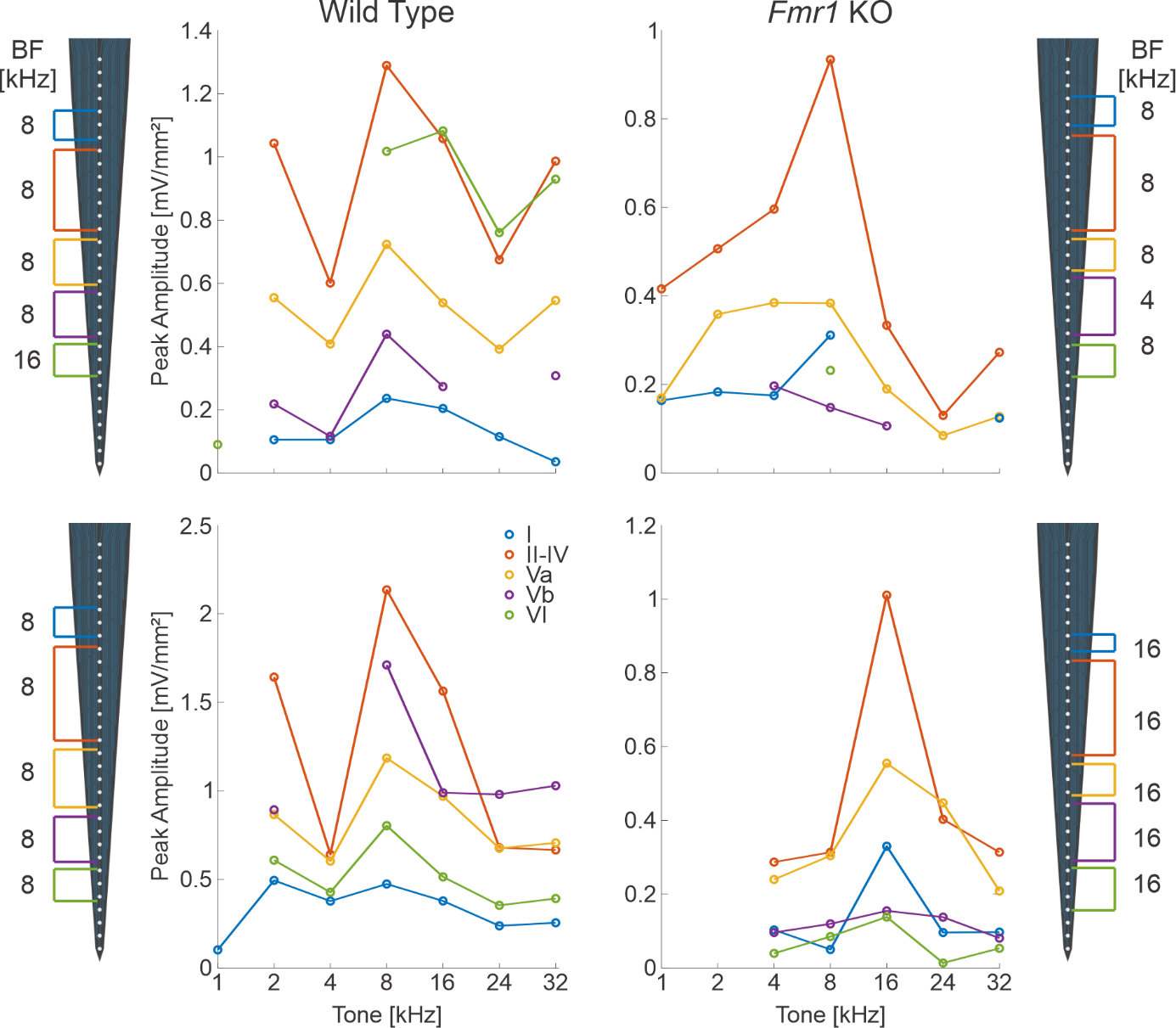


Supplementary Figure 1: Example peak amplitude tuning curves to determine best frequency (BF). Two wild type (left) and two Fmr1 KO (right) example subjects are shown with schematics of the layer coverage over their probe insertion and respective BF for each layer. The lemniscal auditory pathway provides the strongest tuning to input layer IV, with broader tuning and weaker responses in superficial and deep layers. A1 has roughly similar BF responses down layers as determined with LFP/CSD analysis, confirming the perpendicular orientation of the depth electrode.


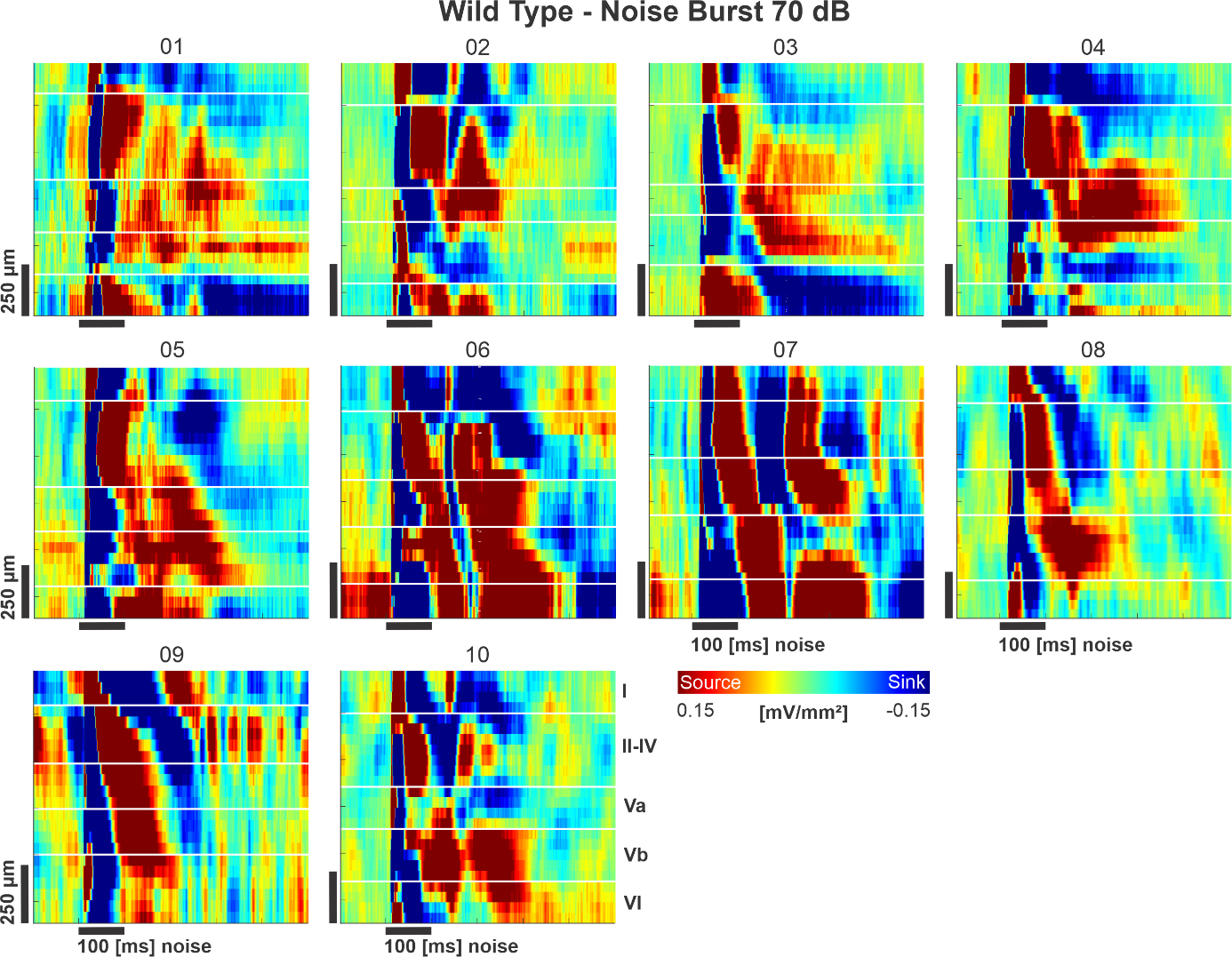


Supplemental Figure 2: Average CSD profiles of WT subjects (n=10), showing neural activity in response to 100 ms noise bursts at 70 dB SPL. Individual layer channel assignments are indicated with white lines (layers I, II-IV, Va, Vb, VI).


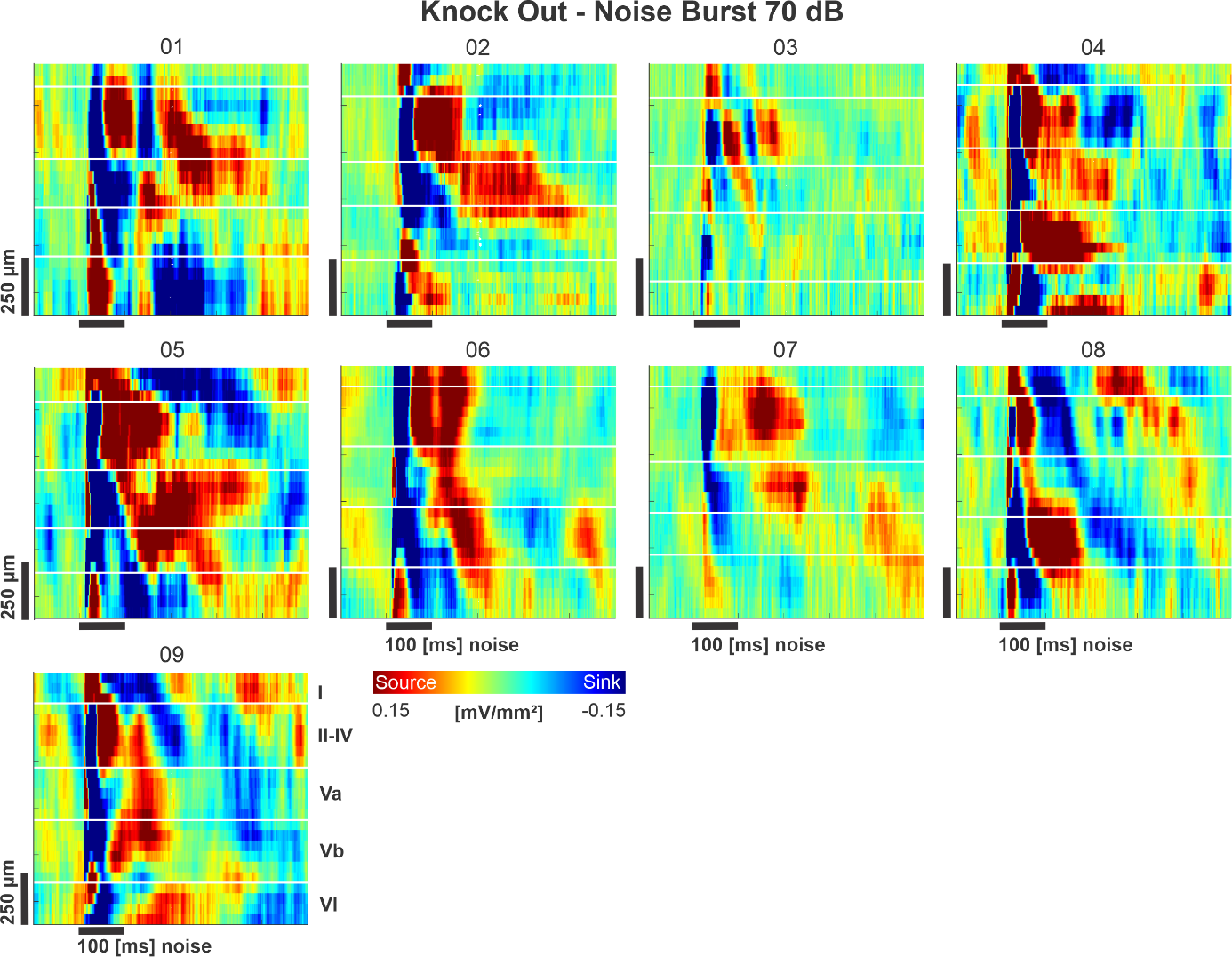


Supplemental Figure 3. Average CSD profiles of Fmr1 KO subjects (n=9), showing neural activity in response to 100 ms noise bursts at 70 dB SPL. Individual layer channel assignments are indicated with white lines (layers I, II-IV, Va, Vb, VI).


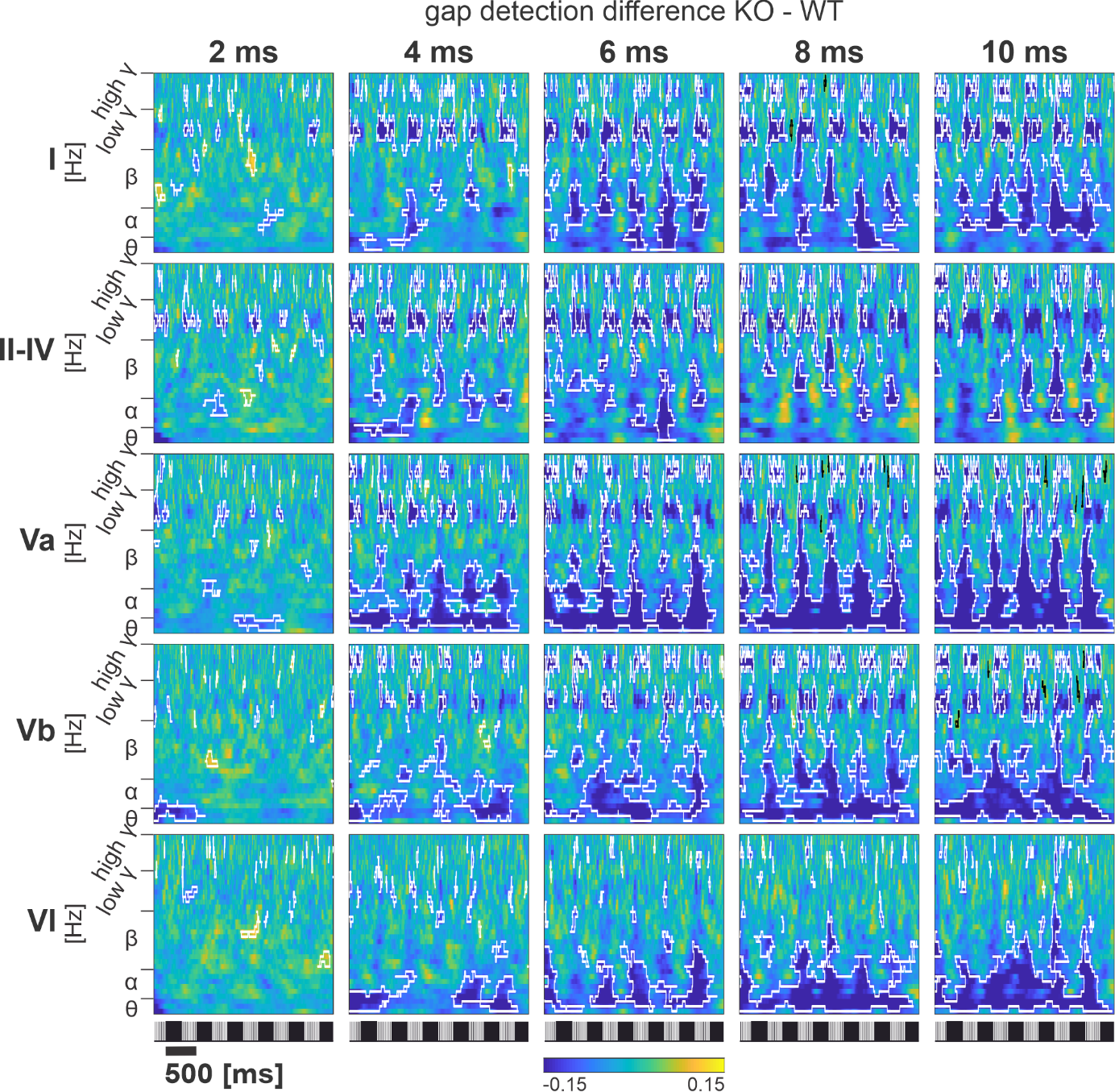


Supplemental Figure 4: Significant gap detection deficit in Fmr1 KO mouse cortical recordings. Group averaged and continuous wavelet transform derived ITPC difference between the Fmr1 KO – WT subjects in response to ~4 second 40 Hz gap ASSR with 75% modulation depth. From left to right: gaps were 2, 4, 6, 8, and 10 ms wide. From top to bottom: layers I-VI. Observed regions of significant difference, by point-wise Student’s t test, are surrounded in white borders (determined by bwboundaries function in Matlab). Significance is verified by Permutation clustermass test with 1000 permutations and only boundaries over 3 pixels high and wide were kept.
